# Folic acid–loaded amino-functionalized MCM-41 as a drug delivery system for cervical cancer modulating apoptotic and survival signals

**DOI:** 10.1038/s41598-026-65500-w

**Published:** 2026-08-13

**Authors:** Rabab K. Khaled, Ahmed A. Abd-Rabou, Magdah Dawy, Mohammed Ahmed Wahba

**Affiliations:** 1 Department of Physical Chemistry, National Research Centre, 33 El Bohouth St. (Former Eltahrir St.), Dokki, Giza, Egypt; 2 Hormones Department, Medical Research and Clinical Studies Institute, National Research Centre, Cairo, Egypt; 3 Stem Cell Lab., Center of Excellence for Advanced Sciences, National Research Centre, Dokki, Giza, 12622 Egypt; 4 Department of Inorganic Chemistry, National Research Centre, 33 El Bohouth St. (Former Eltahrir St.), Dokki, Giza, 12622 Egypt

**Keywords:** MCM-41, Amino functionalization, Folic acid, Drug delivery, Cervical cancer, Apoptotic modulation, Biochemistry, Biotechnology, Cancer, Chemistry, Drug discovery

## Abstract

Cervical cancer remains one of the most prevalent gynecological malignancies worldwide, with high mortality rates particularly in developing countries, necessitating the development of effective and targeted therapeutic strategies. In this study, a folic acid (FA)-loaded amino-functionalized MCM-41 mesoporous silica nanocarrier was developed as a controlled delivery platform for cervical cancer applications. Pure MCM-41 was synthesized via a precipitation method and subsequently functionalized with aminopropyl groups through post-synthesis grafting, followed by FA-loading. Structural, textural and morphological analyses confirmed successful functionalization while maintaining the ordered mesoporous framework. In vitro drug release studies demonstrated pH-responsive behavior, with enhanced folic acid release under physiological conditions (pH 7.4) compared to acidic conditions (pH 1.5), supporting its suitability for controlled delivery applications. Cytotoxicity evaluation using HeLa cervical cancer cells and WI38 normal fibroblasts revealed selective anticancer activity of FA-functionalized systems with minimal toxicity toward normal cells. Among all formulations, the DMSO-based FA-loaded MCM-41 exhibited the highest cytotoxic and apoptotic effects, comparable to Doxorubicin. Mechanistic studies indicated that treatment induced oxidative stress through increased nitric oxide and malondialdehyde levels, accompanied by depletion of antioxidant enzymes (SOD and GSH). Additionally, apoptosis induction was confirmed by an increased Bax/Bcl-2 ratio and activation of the intrinsic mitochondrial pathway, along with suppression of PI3K/AKT survival signaling, along with downregulation of p-AKT. In conclusion, the developed FA-loaded amino-functionalized MCM-41 nanocarrier demonstrates efficient tumor targeting, controlled drug release, and potent apoptosis-mediated anticancer activity, highlighting its potential as a promising platform for cervical cancer therapy.

## Introduction

In recent years, significant research has focused on developing novel drug delivery systems (DDS) that offer distinct advantages over conventional forms. These advancements include enhanced therapeutic efficacy, improved biocompatibility, controlled and prolonged release kinetics that ensure a predictable therapeutic response^1^. Furthermore, some of these systems can serve as a protective system, especially for orally administered active compounds, shielding active ingredients from degradation caused by the highly acidic gastric environment and specific digestive enzymes that would otherwise compromise their biological activity. Folic acid (FA) serves as a prime example of a biological active agent which is highly sensitive to its environment; it degrades in strong acid and loses its biological activity when exposed to light or increases in temperature and pressure^2^.

Folate refers to the naturally occurring form of vitamin B9 found in foods, whereas folic acid is its synthetic and more stable counterpart commonly used in supplements and pharmaceutical formulations. Folic acid is an essential vitamin involved in DNA synthesis and cell proliferation and is mainly used in cancer research as a folate receptor (FR)-targeting ligand rather than a direct chemotherapeutic agent. The mesoporous silica MCM-41 nanocarrier provides a high-surface-area mesoporous structure for efficient loading, while folic acid enhances selective uptake in FR-overexpressing cancer cells, improving overall delivery and anticancer performance^3^. Numerous studies have highlighted its impact on cancer risk and progression. Hwang et al. found that increased folic acid levels can affect S-adenosylmethionine (SAM) concentrations, which in turn influences the expression of cytochrome P450 2E1 (CYP2E1) and lowers the risk of oral cancer^4^. Another investigation explored the link between folic acid consumption and decreased risks of esophageal adenocarcinoma and squamous cell carcinoma in high-risk groups^5^. In the context of lung cancer, various studies have indicated that a higher intake of folic acid, whether from food or supplements, may offer protective benefits^6^. Observational studies have also suggested that sufficient folic acid intake may lower the risk of colon cancer and breast cancer, especially among alcohol consumers^7,8^.

Among these cancer types, cervical cancer ranks as the second most prevalent cancer among women globally and is closely linked to significant morbidity and mortality^9^. The early identification of cervical cancer is crucial, particularly in many developing countries. Without timely detection, it can escalate into one of the most severe gynecological health issues^10^. In these regions, the disease is often diagnosed only in its advanced stages. Patients who delay seeking early diagnosis and treatment frequently experience cancer progression and metastasis^11^. In the United States, numerous cases of cervical cancer are reported each year; unfortunately, nearly half of those affected succumb to this lethal illness^12^. In Egypt, a developing nation, approximately 26 million of women are at risk for cervical cancer^13^.

Therefore, a diverse array of materials has been evaluated as drug delivery systems for biological active agents, including biodegradable polymers^14^, hydrogels^15^, and mesoporous silica^16^. Among them, MCM-41 mesoporous silica has emerged as an optimal candidate for drug delivery system due to its unique structural advantages. Specifically, its high surface area and pore volume facilitate significant drug loading and adsorption. Furthermore, MCM-41 offers tunable morphology^17^, particle size, an ordered pore structure with a narrow size distribution and the possibility to modify the pore size and surface properties in order to control the release of biologically active agents by modulating their interaction with the carrier^18^. These features are complemented by its robust chemical and thermal stability, excellent biocompatibility, and non-toxic nature. In addition, the presence of free silanol groups on the MCM-41 surface allows their functionalization with a diverse range of molecules, which can be tailored to achieve targeted drug delivery and controlled release. Such chemical modifications impart novel properties to MCM-41, enabling targeted drug delivery, controlled release, enhanced bioavailability, and improved cellular uptake^19^. These modifications play a pivotal role in governing drug loading capacity and pharmacokinetic release profiles and their interaction with cells, tissues or molecules. Consequently, the ability to fine-tune these properties makes modified MCM-41 an exceptionally attractive candidate for innovative solutions in both biotechnology and nanomedicine^20^.

The functionalization strategy via post-synthesis grafting of organosilane precursors is widely used due to its procedural simplicity and its ability to anchor diverse molecules or functional groups onto the MCM-41 surface without compromising bulk material properties^21^. For example, the functionalization of mesoporous silica with ligands that are recognized by specific cellular receptor significantly influences both their uptake efficiency and cellular entry pathways, such as by specific bindings with receptors, instead through clathrin-dependent endocytosis mechanism or pinocytosis^22,23^. Numerous studies have highlighted the applications of MCM-41 as a drug delivery system. Luiza et al. investigated folic acid-functionalized mesoporous silica (SBA-15, SBA-16, and MCM-41) as carriers for methotrexate (an anticancer agent), noting that the specific host structure significantly influenced the release kinetics^23^. Simona et al. Synthesized MCM-41 and MCM-41/Fe_3_O_4_ functionalized with folic acid. These systems demonstrated promise as effective carriers for oral drug administration^3^.

Concurrently, in the present study, a pure highly ordered MCM-41, along with folic acid (FA)-loaded amino-functionalized MCM-41, were prepared. FA was employed as a targeting ligand due to its high affinity for folate receptors (FRs), which are overexpressed on the surface of many cancer cells. In addition to facilitating receptor-mediated targeting, the anticancer activity of the FA-loaded amino-functionalized MCM-14 system will be evaluated to assess its potential therapeutic efficacy. The structural, textural and morphological features were also reported. The prepared materials were systematically characterized to confirm successful functionalization, evaluate structural integrity, and assess changes in surface and pore properties following modification. In addition, the functionalized systems were investigated for their drug delivery performance and biological activity to establish their potential as targeted nanocarriers for cervical cancer therapy.

## Experimental

### Materials and reagents

Hexa decyl trimethyl-ammonium bromide (CTAB, C_19_H_42_Br N), MW = 364.45, Acros Organic, Belgium. Sodium silicate (Na_2_SiO_3_), MW = 122.06. Sodium hydroxide pellets (NaOH), MW = 40 g/mol, SIGMA-ALDRICH. Folic acid, Escpharmceutical Chemicals Co. (3-Amino propyl) tri-ethoxysilane (H_2_N (CH_2_)_3_ Si (OCH_2_CH_3_)_3_, 98%), FW = 221.37, Alfa Aesar, ThermoFisher, Germany. Dimethylsulfoxide (C_2_H_6_OS, DMSO), MW = 78.13, Research-Lab Fine Chem Industries, India.

**Figure:**
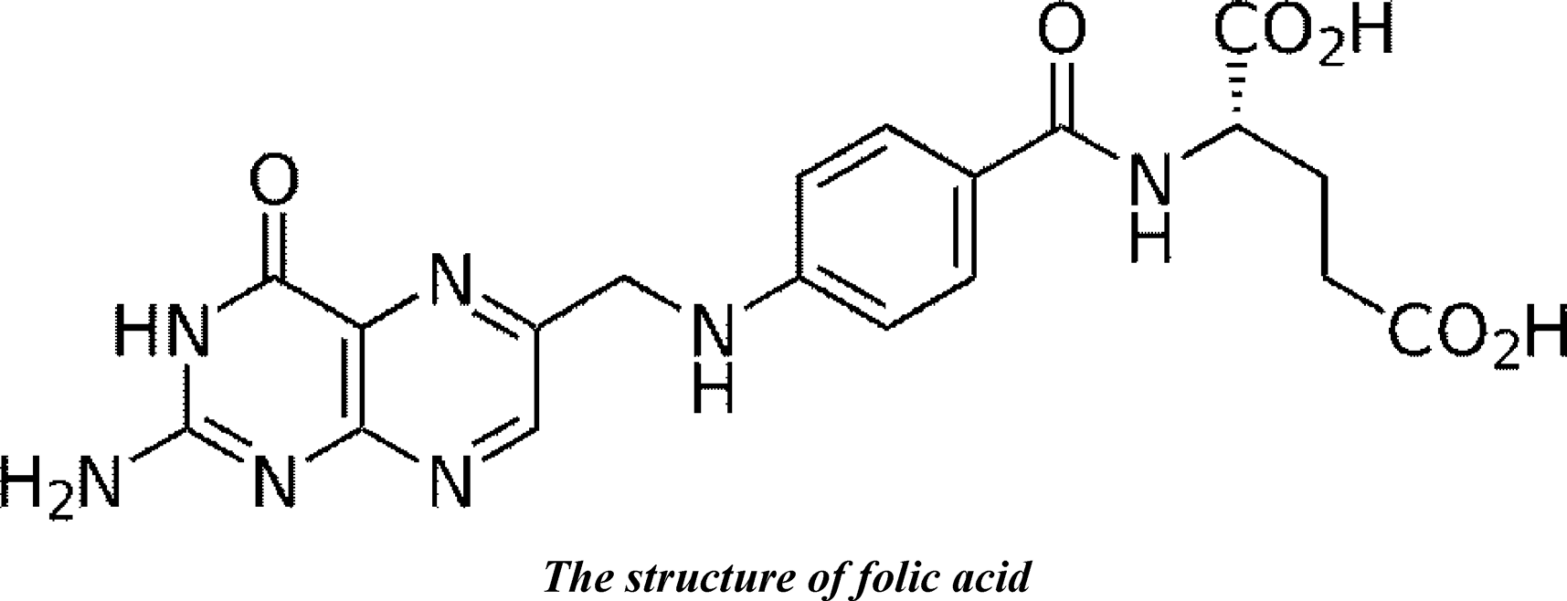

### Preparation of pure MCM-41 by precipitation method

3.64 g CTAB was dissolved in NaOH solution to form a homogeneous mixture. Subsequently, sodium silicate solution (10.16 g Na_2_SiO_3_ in 80 mL distilled water) was added dropwise, and the pH was adjusted to 10. The reaction mixture was stirred at room temperature for 24 h, followed by filtration, washing, and drying at 70 °C. The final product was calcined at 550 °C for 6 h to obtain pure MCM-41, denoted as M sample^24^.

### Functionalization of MCM-41 by post-synthesis grafting method

Amine-functionalized MCM-41 was achieved using 3-Amino propyl tri-ethoxysilane (APTES) as a bifunctional organosilane widely used as a surface coupling agent to introduce amino groups onto silica materials, according to the following procedure. 1.0 g of MCM-41 was stirred vigorously in 60 ml toluene containing 10 mmol of (3-Amino propyl) tri-ethoxysilane. This solution was refluxed at 120 °C for 24 h. The powder was collected by filtration, washed with ethanol and distilled water, and finally dried at 60 °C. The obtained amino functionalized MCM-41 was designated as AM sample^25^.

### Folic acid loading into amino-MCM-41

The folic acid-loaded amino-functionalized MCM-41 was prepared by allowing FA to associate with amino- functionalized MCM-41 through surface interactions during the loading process. In a typical method, FA (62.50 mg) was dissolved in 50 mL of dimethylsulfoxide (DMSO). 50 mL of DMSO containing 0.25 gm of amino-MCM-41 was added and allowed to react for 24 h under reflux at 80 °C. The FA-loaded amino-functionalized MCM-41 was separated by centrifugation. Then FA- loaded NH_2_-MCM-41 was dried at 60 °C and denoted as FAMD. The same procedures were done except utilizing distilled water instead of DMSO and this sample coded as FAMW.

### Characterization

N_2_ adsorption–desorption isotherms were measured at  −196 °C using a NOVA 3200 instrument (USA) to calculate specific surface area (S_BET_), pore size distribution (using BJH method), and pore volume. Initially, the samples were degassed under vacuum (10^−4^ Torr) at 120 °C overnight. The particles morphology and size were analyzed using a transmission electron microscope (TEM, JEOL JEM-2100). The functional groups present in the samples were identified through FTIR transmittance spectra using a Brucker Vertex 80 V from Bruker Optik GmbH, Germany, using KBr pellet method in the range of 4000–400 cm^−1^ with the resolution of 4 cm^−1^. The FA loading and amino functionalization of MCM-41 were further confirmed using the CHN elemental analysis to determine the nitrogen concentration values.

### Drug delivery study

The drug delivery profiles of folic acid were approached according to a previous protocol^3^, with some modifications. 0.01 g mesoporous folic acid delivery systems (folic acid-amino-MCM-41-water FAMW and folic acid-amino-MCM-41-DMSO FAMD) were individually suspended in 50 mL simulated body fluid (SBF) or hydrochloric acid solution (pH ~ 1.5), under mechanical stirring, at room temperature. At certain times (0 h, 4 h, 8 h, 12 h, 24 h, 48 h, and 72 h), 1 mL of each solution were taken and filtered through a 0.25 μm Agilent Econofilter syringe filter membrane and used for quantification. The calibration curve of the standard folic acid was performed. The spectra of folic acid displayed a strong absorption band at 286 nm^26^.

### In vitro experiments

#### Cell culture and maintenance

Cervical cancer HeLa cells and normal WI38 cells were purchased from American ATCC Company, USA. Cells were cultured using DMEM medium. Medium was supplemented with 4.50 g/L glucose and L-glutamine and 10% FBS. Cells were incubated at 5% CO_2_ humidified at 37 °C for growth maintenance.

#### Cellular uptake study

Cellular uptake of the formulations was evaluated in human cervical carcinoma HeLa cells (FRα-positive) and normal human lung fibroblast WI-38 cells (FRα-negative). In this study, the folic acid (FA) moiety is consistently defined as a surface-conjugated targeting ligand responsible for folate receptor-mediated uptake, rather than as a free therapeutic agent. Cells were cultured under standard conditions (37 °C, 5% CO_2_) and seeded in 6-well plates at an appropriate density prior to the experiment. To confirm folate receptor-α (FRα) expression, cells were incubated with Alexa Fluor™ 488-conjugated Anti-Human Folate Receptor Alpha (FR1/FRα) Monoclonal Antibody (BioLegend, USA) according to the manufacturer’s instructions. Following incubation, cells were washed with phosphate-buffered saline (PBS) and analyzed for receptor expression. For the uptake study, HeLa and WI-38 cells were incubated with the fluorescently labeled formulations: F, FAMW, and FAMD. At time intervals (0, 4, 8, 12, and 24 h for HeLa cells; 0, 4, and 24 h for WI-38 cells), cells were harvested, washed three times with cold PBS to remove non-internalized particles, and resuspended in PBS for analysis. Quantitative cellular uptake was determined using a BD Flow Cytometry Biosciences (USA). Data were analyzed using FlowJo software.

#### Cytotoxicity using MTT assay

To test the cytotoxicity of the formulas folic acid-amino-MCM-41-water (FAMW) and folic acid-amino-MCM-41-DMSO (FAMD), in addition to pure MCM-41 (M), amino-MCM-41 (AM), and folic acid (F) against folic acid receptor positive (FR^+^) HeLa cells and folic acid receptor negative (FR^−^) WI38 cells. This technique was approached using MTT assay^27^. The cells were extracted by tripsinization, cleaned in phosphate buffer saline, and plated with 96-well plates before testing. For 24 h, the cells were cultured at 37°Cwith 5% CO_2_. Following a 24 h incubation period, the cells were exposed to 0, 5, 10, 20, 40, 80, 120, 240 and 480 µg/mL of the proposed formulas and the doxourubicin (DOX), as a positive control. The ability of living cells to convert the yellow dye 3-(4,5-dimethyl-2-thiazolyl)-2,5-diphenyl-2H-terazolium bromide (MTT) into a blue formazan product is used to measure the cell viability % at 540 nm using an ELISA reader.

#### Flow cytometry (Annexin V–PI) assay

Apoptosis was analyzed in HeLa and WI38 cells using Annexin V–FITC/PI staining followed by flow cytometry (BD Biosciences, USA). Cells were cultured under standard conditions and treated with folic acid (F), FAMW, FAMD, and doxorubicin (DOX) at a moderate concentration of 80 µg/mL for 24 h. This concentration was selected as it represents the midpoint of the dose range, where preliminary cytotoxicity data showed the onset of significant biological response without complete cell death, allowing accurate discrimination between viable and apoptotic populations. After treatment, cells were harvested, washed with PBS, and stained with Annexin V–FITC and PI in binding buffer for 15 min in the dark. Samples were then analyzed by flow cytometry and data were analyzed using FlowJo software.

#### Assessment of dose-dependent oxidative stress and apoptosis

The levels of lipid peroxidation and oxidative stress were evaluated by measuring malondialdehyde (MDA) and nitric oxide (NO) using spectrophotometric assay kits purchased from Biodiagnostic® (Egypt) according to the manufacturer’s instructions. The assays were performed using a colorimetric method at 540 nm. HeLa cells were treated with low (20 µg/mL), moderate (80 µg/mL), and high (240 µg/mL) doses of F, FAMW, FAMD, and DOX to investigate dose-dependent effects on oxidative stress markers. In addition, dose-dependent apoptosis was evaluated using Annexin V–FITC/PI flow cytometry following treatment with the corresponding low and high doses. Samples were then analyzed by flow cytometry and data were analyzed using FlowJo software.

#### Antioxidant enzymes

Superoxide dismutase (SOD) and glutathione (GSH) activity levels were assayed according to ELISA kits purchased from Elabscience (China) and they were performed according to manufacturer instructions. These enzymes were measured using colorimetric method at 450 nm. HeLa cells were treated with low (20 µg/mL), moderate (80 µg/mL), and high (240 µg/mL) doses of F, FAMW, FAMD, and DOX to investigate dose-dependent effects on the antioxidant enzymes.

#### Expression levels of apoptotic and survival proteins

Apoptotic (Bax and Bcl-2) and survival (PI3K, AKT, and phosphorylated AKT) protein expression levels were assayed using ELISA kits purchased from Sunlong (China) according to the manufacturer’s instructions. The measurements were performed using a colorimetric method at 450 nm.

### Statistics

A one-way ANOVA was conducted, followed by the post-hoc test, to determine significant differences between treated cells and untreated control cells. The data are presented as mean ± standard deviation. a; represents a statistical difference (*p* < 0.05) and b; represents a high statistical difference (*p* < 0.01).

## Results and discussion

### FT-IR

**Fig. 1:**
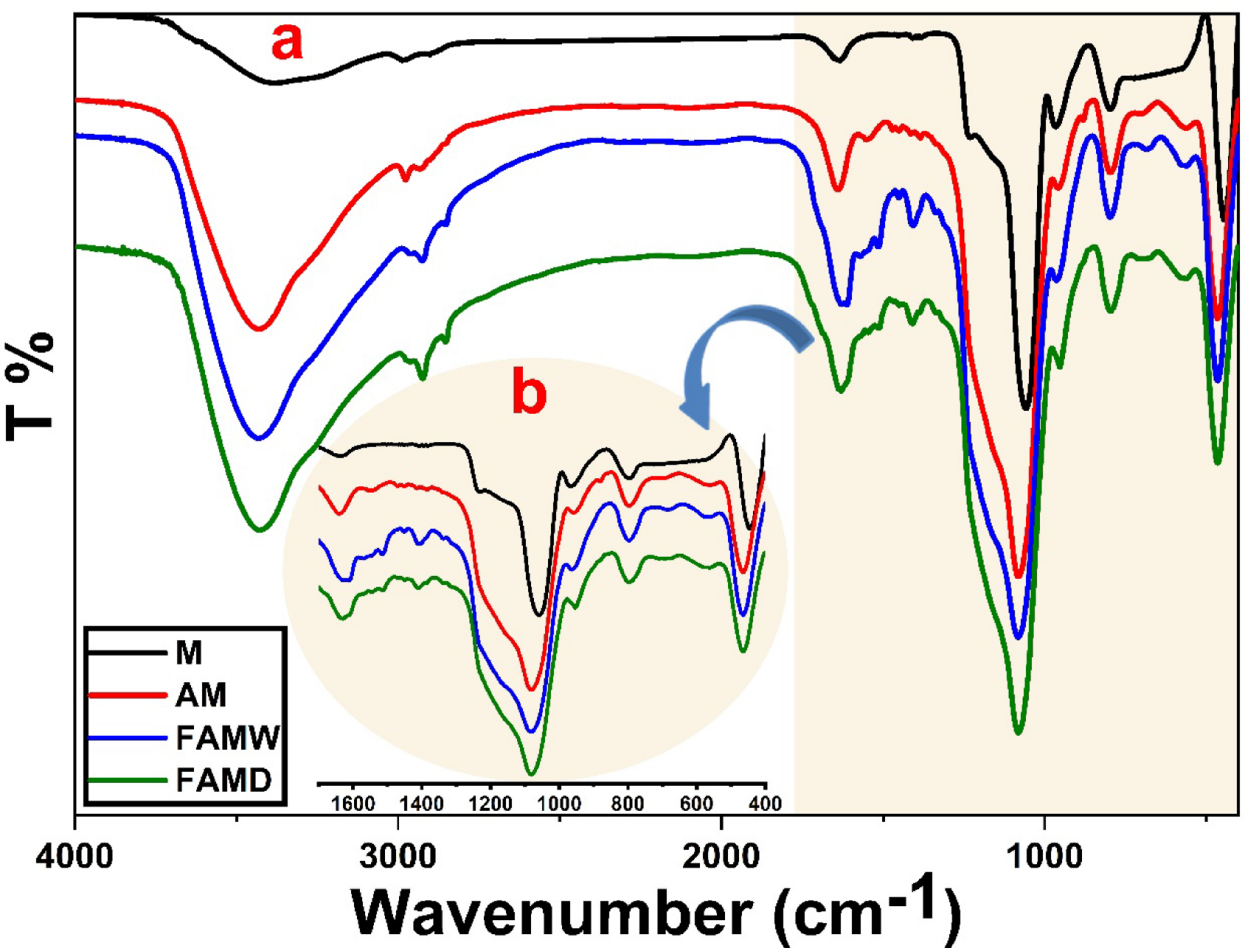
(**a**) FT-IR spectra (400–4000 cm^− 1^) of M, AM, FAMW and FAMD samples and (**b**) Magnified FT-IR spectra in the (400–1600 cm^− 1^) range.

IR spectra of pristine MCM-41 and functionalized samples are displayed in Fig. 1. For a more detailed view of the vibrational bands, the inset in Fig. 1b shows a zoomed-in region between 400 and 1600 cm^−1^. The overall spectral features in the 400–4000 cm^−1^ range are distinctive of the MCM-41 framework^28–30^. Within the pure MCM-41 sample, a broad band is detected at 3379.50 cm^−1^ attributed to hydroxyl stretching (O–H) groups, influenced by H_2_O_ads_ ; additionally, the 1637.70 cm^−1^ peak belongs to the H_2_O bending mode^30,31^. Asymmetric υ Si–O-Si are observed at 1227.8 and 1058.9 cm^−1^, while symmetric Si–OH stretching is indicated by the 963.6 cm^−1^ band. While the (Si–O-Si)_sym_ stretching is represented by the 794.70 cm^−1^, and the discrete band at 446 cm^−1^ corresponds to the siloxane υ_bending_ Si–O-Si^32^. Upon FA loading and amino functionalization, several notable changes and presence of new bands were observed. For AM, FAMW and FAMD samples (Fig. 1), the 3379.50 band transmittance intensity increase, with shifts to 3430.50 cm^−1^ in AM, 3430.50 in FAMW and 3430.50 in FAMD. The bands around 1630 cm^−1^ and 1550 cm^−1^ are assigned to the CO amide I and NH amide II groups. This intensity and shifts variations are consistent with the presence of additional organic moieties and changes in the surface chemical environment after modification, in agreement with previous reports, aligning with prior reports^33,34^. Presence of N–H bending vibration at 564.20 cm^−1^ for all functionalized samples and NH_2_ symmetric bending vibration at 1547.70 cm^−1^ for AM and 1513.90 cm^−1^ for FAMW and FAMD samples, absent in neat MCM-41, indicates the introduction of nitrogen-containing organic species onto the material^33,35^. The bands in the 3000–2800 cm^−1^ range are due to the asymmetric and symmetric stretching modes of CH_2_ groups of silane introduced onto the silica surface by the grafting procedure^36^. Specifically, the υ_stretching_ Si–O–Si at 1058.90 cm^−1^ in pure M was shifted to 1081.5 cm^−1^ in all functionalized samples. Additionally, alterations in the intensity and position of the Si–OH stretching band at 963.6 cm^−1^ shifting to 957.60 cm^−1^ with AM, 963.60 cm^−1^ with FAMW and 952.30 cm^−1^ with FAMD suggest modification of the surface silanol environment changes following FA loading and amino functionalization^23^.

### TEM analysis

**Fig. 2:**
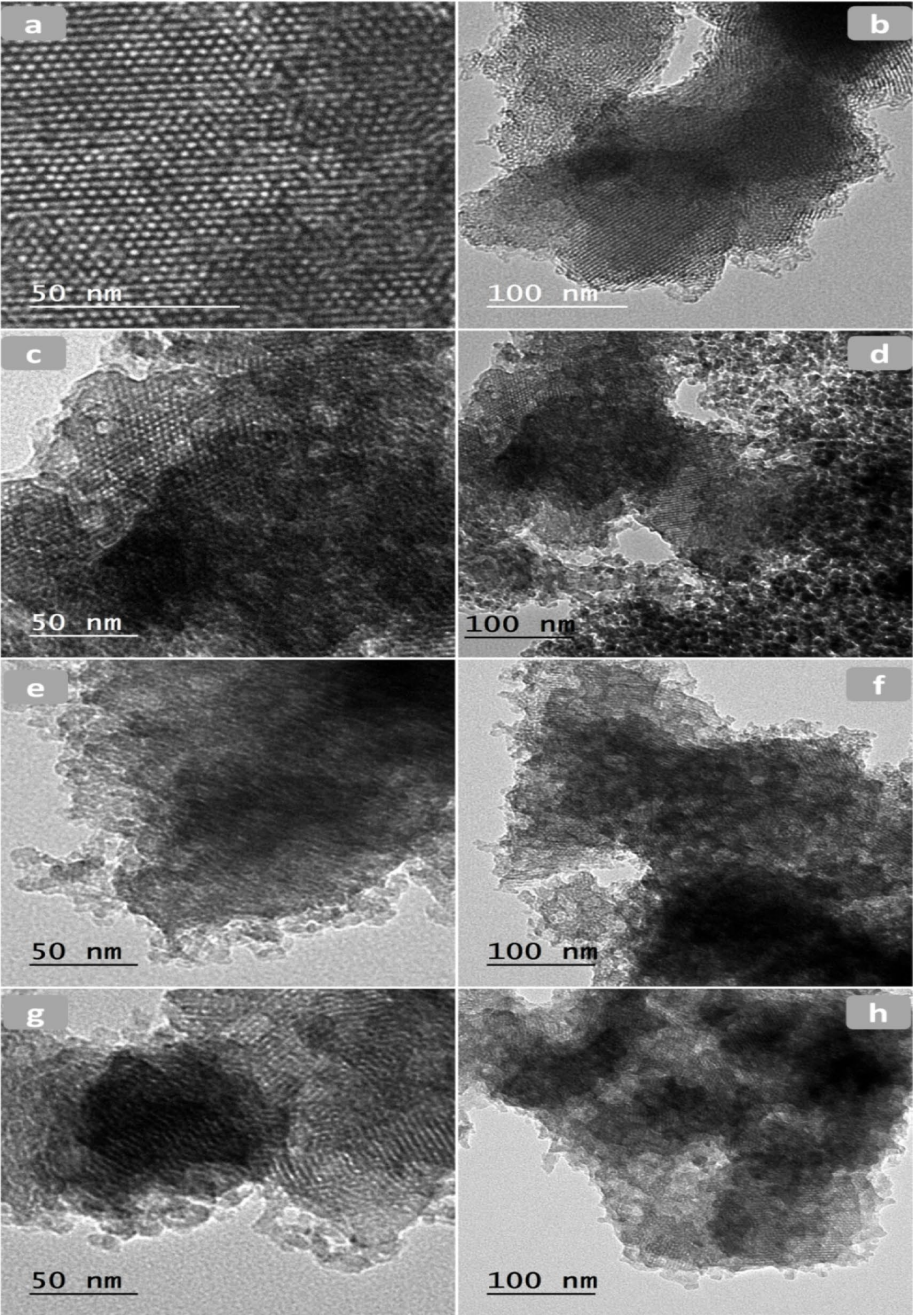
HR-TEM images of (**a**, **b**) M, (**c**, **d**) AM, (**e**, **f**) FAMW and (**g**, **h**) FAMD samples.

TEM images indicate that the mesostructural features of MCM-41 were largely retained after amino functionalization and loading of folic acid. Figure 2 shows the typical channels of the mesoporous matrixes as pseudo hexagonal arrays of pore voids. These channels were visualized not only in pure MCM-41 but also in AM, FAMW and FAMD, suggesting the preservation of the ordered mesoporous architecture after modification. The TEM image of pure M (Fig. 2a) showed a distinct hexagonal pore pattern, where each pore is encircled by 6-adjacent pores. A uniform linear pattern aligned vertically along the pore channels is displayed in Fig. 2b, confirming a highly ordered arrangement^32^. The TEM images of functionalized samples (Fig. 2c–h) detected a pore structure similar to M sample, although some irregularities and defects, darker and high-contrast spots or clusters are present, which may be associated with surface modification and changes in local textural features.

### Elemental analysis

The organic functionalization of the MCM-41 mesoporous framework was quantitatively verified through C, H, N elemental analysis, with results summarized in Table 1. The emergence of nitrogen content in the modified samples, absent in the bare silica matrix (MCM-41), provides definitive evidence of the successful grafting of aminopropyl groups and the subsequent loaded of folic acid. Theoretically, the C/N ratio for complete hydrolysis of ethoxy groups is 3. For AM sample, the C/N ratio is 4.99, the elevated carbon content points toward the retention of unhydrolyzed ethoxy groups from the APTES precursor. Such steric hindrances or incomplete condensation are common in anhydrous grafting environments, where the density of surface silanols and the availability of water limit the displacement of alkoxy groups^33,37^. Following the loading with folic acid (FAMW and FAMD samples), the C/N ratio decline to 3.99 and 4.10, respectively. The decrease relative to the AM sample is indicative of successful folic acid attachment. Folic acid is a nitrogen-rich molecule containing seven nitrogen atoms per molecule. Its covalent attachment via amide bond formation significantly increases the nitrogen weight percentage of the total organic mass, thereby pulling the C/N ratio downward.

**Table 1:** C, H, N elemental analysis of AM, FAMW and FAMD samples.

Samples	C%	H%	N%	C/N
AM	30.13	4.25	6.06	4.97
FAMW	37.15	3.89	9.31	3.99
FAMD	33.46	3.57	8.12	4.10

### BET analysis

**Fig. 3:**
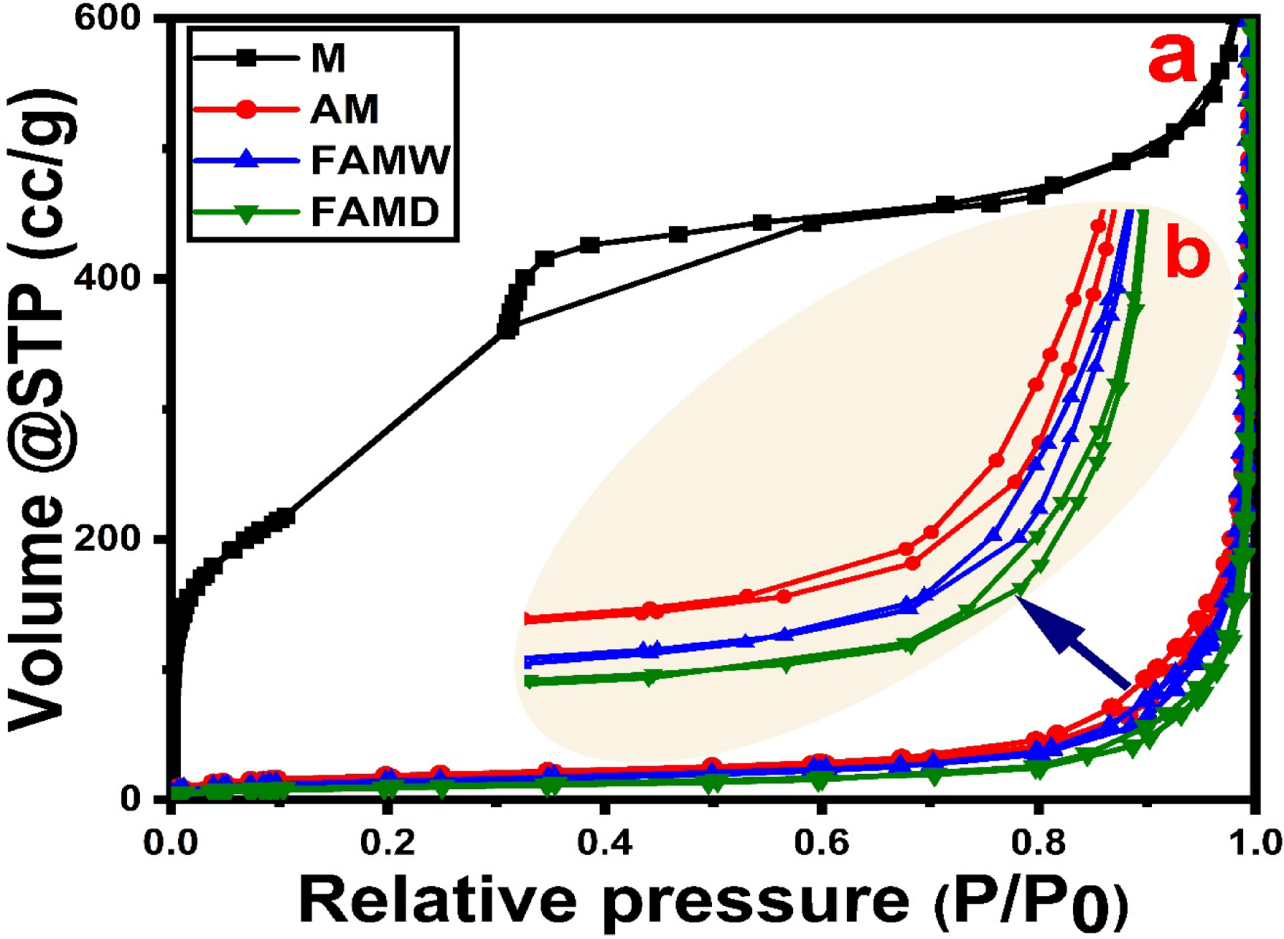
(**a**) N2 adsorption–desorption isotherms of M, AM, FAMW and FAMD samples and (**b**) Zoomed view of p/p0 range from 0.6 to 1 for AM, FAMW and FAMD samples.

Figure 3 illustrates the complete N_2_ adsorption–desorption isotherms for all samples across P/P_0_ range of (0 to 1.00). All the synthesized materials exhibit characteristic type IV isotherms based on the IUPAC classification^38^. For pure M sample (Fig. 3a), a characteristic hysteresis loop was observed in the range of 0.30–0.60 attributed to capillary condensation within the mesopores. Following a stable adsorption region between relative pressures of 0.60 and 0.80, the curve rises sharply at higher pressures (0.80 < P/P_0_ < 1.0); which may reflect interparticle textural porosity and multilayer adsorption on the external surface rather than uniform filling of the intrinsic mesopores. The lack of a plateau as the relative pressure approaches 1.0 suggests that the adsorption saturation was not reached. This sample exhibited a high BET-surface area of 1100 m^2^/g ^24^. Compared to their parent MCM-41, AM, FAMW and FAMD samples display distinct differences in N_2_ adsorption, especially in the multilayer zone. These variations arise from altered surface properties. In particular, the isotherms lack distinct steps at low-to-intermediate relative pressures, reflecting a lower maximum adsorbed volume. This trend aligns with the observed decrease in BET surface area after FA loading and amine functionalization and are consistent with partial pore filling, surface coverage by organic moieties, and increased textural heterogeneity after functionalization^39^.

For all functionalized samples (Fig. 3a), at low relative pressure (0 to 0.1), the curve shows a slight "knee," representing the formation of a monolayer. At P/P_0_ (0.10 to 0.80), there is a gradual linear representing the buildup of multiple layers of adsorbate on the pore walls. In Fig. 3a&b, the slope associated with the capillary condensation, in the P/P_0_ ranges of AM 0.60–0.97, FAMW 0.80–0.97, and FAMD 0.80–0.97, is less pronounced for amino and FA-amino functionalized MCM-41 samples than for the pure MCM-41. This suggests a broader distribution of pore diameters in amino and FA-amino functionalized samples. At higher relative pressures, there is an increase in adsorption in amino and FA-amino functionalized samples due to multilayers adsorption on the mesoprous exterior surface. The type of hysteresis loop observed is indicative of the pore network’s influence. Furthermore, AM, FAMW and FAMD samples display an H3 hysteresis loop, this suggests the presence of narrow, slit-like mesopores in the materials^40,41^.

**Fig. 4:**
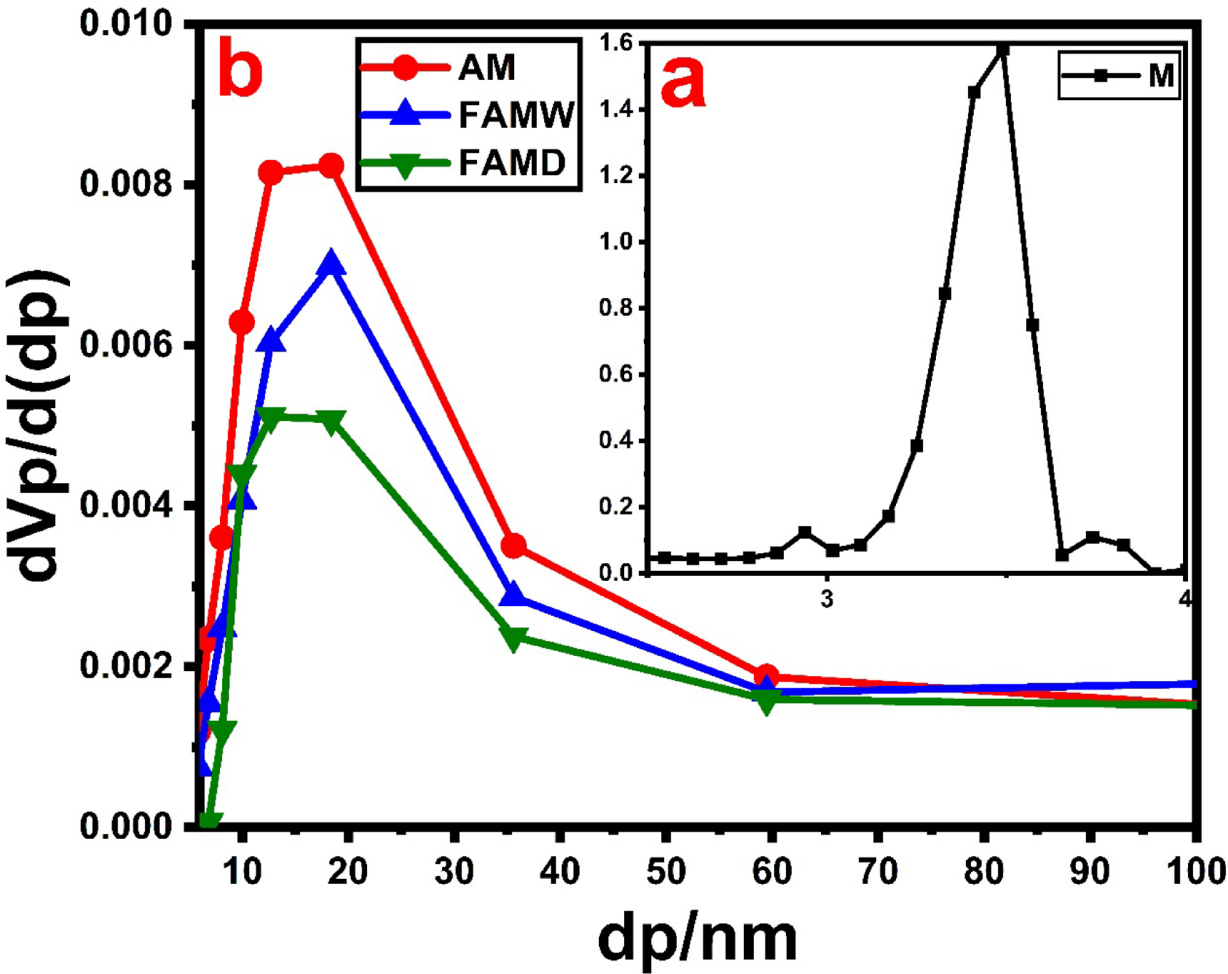
Pore size distribution of (**a**) M and (**b**) AM, FAMW and FAMD samples.

Figure 4 displays the BJH pore size distribution for unmodified as well as functionalized MCM-41 samples. The pure sample exhibits a confined mesopore size distribution, while amino and FA-amino functionalized samples show a broader distribution of pore diameters. However, these apparent changes should be interpreted cautiously, since BJH analysis may become less reliable for heavily modified mesoporous materials due to pore blocking, network effects, and changes in adsorption/desorption behavior. Therefore, the less uniform distribution of pore sizes in the functionalized MCM-41 samples are more appropriately taken as an indication of increased textural heterogeneity and reduced uniform pore accessibility after functionalization, rather than definitive evidence of true pore enlargement. This may be potentially stemming from irregularly arranged pores and/or a higher defects concentration in the pore walls. The broad tail extending to larger calculated pore widths may also reflect interparticle voids and textural porosity.

Table 2 presents an overview of the textural properties of the synthesized materials determined from adsorption–desorption isotherms analysis. After FA loading and amine functionalization, a marked decrease was observed in specific surface area and pore volume (Fig. 3 and Table 2). M sample exhibited BET surface area of 1100 m^2^/g, that was reduced to 70.67, 53.10, and 35.40 m^2^/g in the AM, FAMW, and FAMD samples, respectively. Also, the pore volume of M sample reduced from 1.15 cm^3^/g to 0.41, 0.37 and 0.29 cm^3^/g for AM, FAMW and FAMD samples, respectively. These observed facts evidence that the pore wall is indeed covered by the organic moieties (aminopropyl group and FA) and also, the organic moieties located within the pores of the matrices, which leads (as expected) to less available space for adsorbed nitrogen and fills the pore voids and this confirms the FA loading and amine functionalization onto MCM-41^42^. In contrast, the apparent increase in BJH pore diameter should not be interpreted as definitive pore enlargement, but rather as a calculated effect associated with broadened pore size distribution and altered adsorption behavior after functionalization. This interpretation is qualitatively consistent with the TEM observation^43^. Similar behavior was observed for other organic functionalized mesoporous silicas^44,45^. The combined FTIR and elemental analysis results support successful amino functionalization and folic acid loading/association on the MCM-41 surface; however, definitive confirmation of covalent bonding requires additional analyses such as XPS or TGA however, these measurements are not available at this stage.

**Table 2:** Textural properties of M, AM, FAMW and FAMD samples.

Samples	N_2_ adsorption–desorption isotherms		
	S_BET_ (m^2^.g^-1^)	V_p_ (cm^3^/g) BJH	D_p_ (nm) BJH
M	1100.00	1.15	3.40
AM	70.67	0.41	23.10
FAMW	53.10	0.37	27.56
FAMD	35.40	0.29	32.80

S_BET_: surface area; V_p_: pore volume; D_p_: average pore size, calculated by BJH model.

### Drug delivery

The release profiles are essential in proving the functionality of FAMW and FAMD formulas in drug delivery. In this study, folic acid is consistently considered as the loaded therapeutic payload within the mesoporous MCM-41 structure, while the carrier system is responsible for protection and controlled release. The two formulas loaded with folic acid were tested in two relevant environments: hydrochloric acid solution (pH 1.5—mimicking the gastric fluid) and SBF (pH 7.4—mimicking intestinal fluid, blood, etc.) over a period of time of 72 h^46^.

**Fig. 5:**
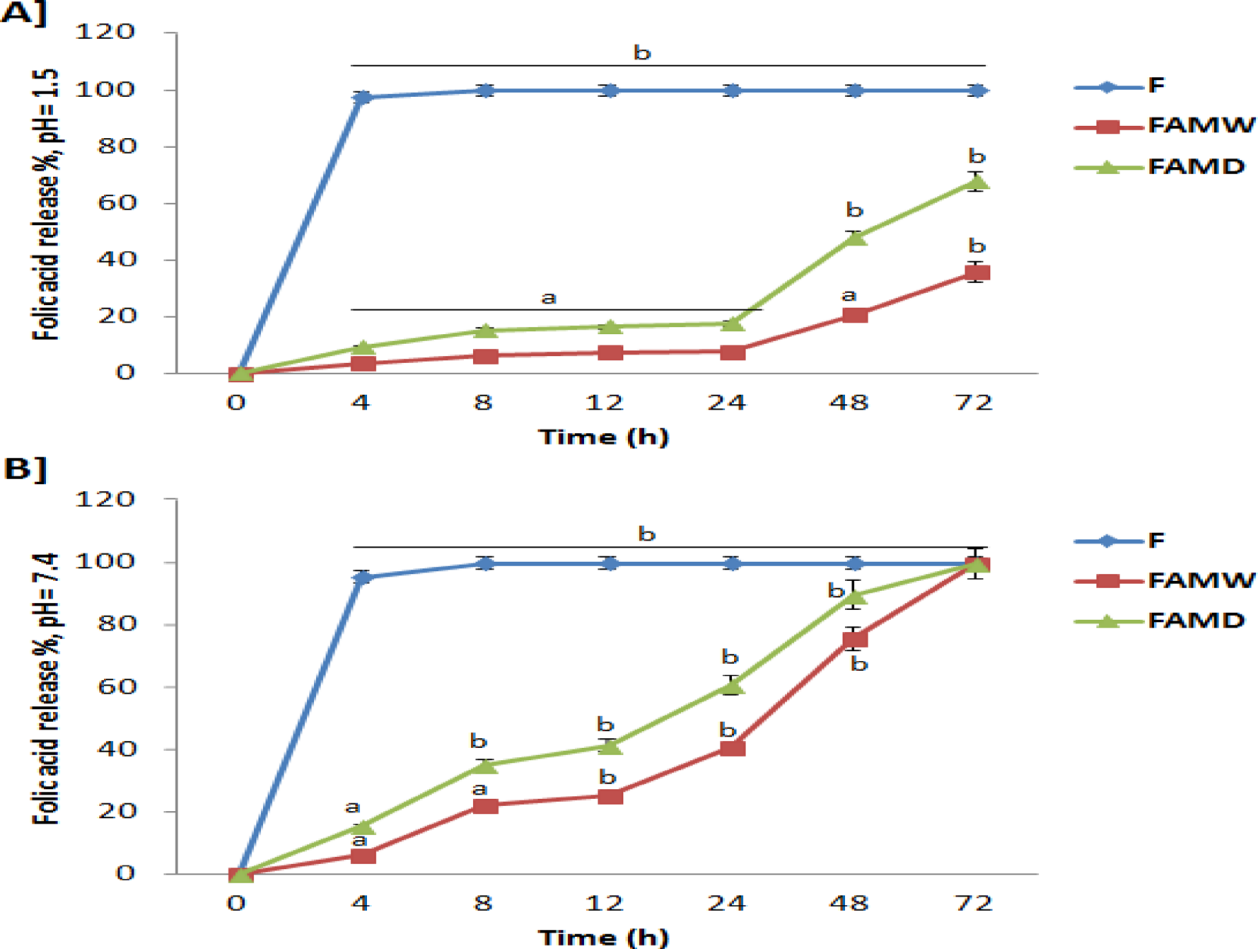
Release profiles of folic acid from FAMW and FAMD at pH = 1.5 (**A**) and pH = 7.4 (**B**).

The two systems were found to exhibit very different release behavior in both environments (Fig. 5 A, B). At the acidic medium (pH = 1.5), the release of folic acid from the FAMW and FAMD was illustrated in (Fig. 5A). After 4 h of release, 3.5% and 9.5% of folic acid were released from FAMW and FAMD, respectively, while the free folic acid showed a burst release of 97.5%. After 8 h, the cumulative release reached 6.3% and 15.3% for FAMW and FAMD, respectively, whereas free folic acid reached complete release (100%). At 12 h, only a slight increase was observed for the loaded systems, reaching 7.5% and 16.5% for FAMW and FAMD, respectively, while free folic acid remained fully released. After 24 h, the release remained relatively stable for the formulations, reaching 7.8% and 17.8% for FAMW and FAMD, respectively. At extended time points, a slow gradual increase was observed, reaching 20.8% and 47.8% at 48 h, and 35.8% and 67.8% at 72 h for FAMW and FAMD, respectively, while free folic acid remained constant at 100% throughout the experiment. This indicates that both formulations significantly suppress folic acid release in acidic conditions, with FAMW exhibiting stronger retention than FAMD.

On the other hand, under neutral conditions (pH = 7.4), a markedly enhanced and time-dependent release behavior was observed for both systems (Fig. 5B). After 4 h, 6.5% and 15.5% of folic acid were released from FAMW and FAMD, respectively, compared to 95.5% release of free folic acid. At 8 h, the release increased to 22.3% and 35.3% for FAMW and FAMD, respectively. At 12 h, the values further increased to 25.5% and 41.5%, and reached 40.8% and 60.8% after 24 h for FAMW and FAMD, respectively. A continued sustained release profile was observed over longer periods, reaching 75.8% and 89.8% at 48 h, and approaching nearly complete release at 72 h with 99.8% for both formulations, indicating complete drug liberation under physiological conditions.

It was clear that the release of the folic acid from the FAMD was higher than FAMW. This behavior may be attributed to differences in the strength of non-covalent interactions between folic acid and the amino-functionalized MCM-41 surface, together with medium-dependent solvation and diffusion effects that influence folic acid desorption and release. In addition, differences in solvent-mediated host–guest interactions may contribute to weaker folic acid retention in the DMSO-prepared system, resulting in a faster release profile. Furthermore, differences in hydrogen bonding networks and surface accessibility of the functional groups may also play a significant role in modulating the release behavior between the two systems.

The folic acid release profiles from FAMW and FAMD demonstrated a clear pH-responsive and time-dependent behavior, consistent with the diffusion/desorption of folic acid associated with the amino-functionalized mesoporous carrier. Both formulations showed significantly higher release at physiological pH (7.4) compared to acidic conditions (pH 1.5), indicating stability in acidic environments and enhanced drug diffusion under biologically relevant conditions. Such pH-dependent release behavior is a well-recognized characteristic of mesoporous silica-based carriers and is considered highly advantageous for oral and tumor-targeted delivery systems, as it minimizes premature drug loss in gastric conditions while promoting release in systemic environments^47,48^.

Among the two formulations, FAMD exhibited a consistently higher cumulative release than FAMW at all-time points, reaching 60.90% after 24 h compared to 40.80% for FAMW. This difference can be attributed to variations in drug–carrier interactions influenced by the loading medium, where DMSO promotes more favorable dispersion and weaker hydrogen bonding between folic acid and surface functional groups, resulting in enhanced diffusion and faster release. Similar effects of solvent polarity on drug loading efficiency and release kinetics in mesoporous silica systems have been previously reported^49,50^. The observed release behavior is therefore more consistent with the reversible association of folic acid within/on the amino-functionalized mesoporous carrier than with cleavage of a confirmed stable covalent linkage.

Overall, both systems demonstrated controlled and sustained release profiles, with FAMD showing superior performance, highlighting its potential as a more efficient folic acid delivery platform for targeted cancer therapy^50^.

#### Kinetic modeling for drug release profiles

Zero-order model (Qt = Q0 + k0t)

Qt is the cumulative amount of drug released at time *t*, Q0 is the initial amount of drug in solution (usually zero), and k0 is the zero-order release constant. The zero-order model assumes a constant drug release rate independent of drug concentration. As shown in Table 3, the free folic acid (F) formulation exhibited poor agreement with the zero-order model at both pH 1.5 and pH 7.4 (R^2^ = 0.550 and 0.530, respectively), reflecting its rapid burst release profile. In contrast, FAMW and FAMD showed better linearity, with R^2^ values ranging from 0.940 to 0.990. The zero-order release constants (k0) increased from 0.48 to 1.39 h⁻^1^ for FAMW and from 0.96 to 1.71 h⁻^1^ for FAMD as the pH increased from 1.5 to 7.4, indicating faster release under physiological conditions. Nevertheless, the lower goodness-of-fit compared with the diffusion-based models suggests that drug release was not governed by a constant release rate throughout the experimental period.

**Table 3:** Release kinetic model parameters for formulations at pH 1.5 and pH 7.4.

Formulation	pH	Zero-order k (h⁻^1^)	R^2^	First-order k (h⁻^1^)	R^2^	Higuchi k (h⁻½)	R^2^	Korsmeyer–Peppas k	n	R^2^
F	1.5	2.09	0.550	0.910	0.999	37.20	0.940	0.340	0.18	0.998
	7.4	2.08	0.530	0.770	0.997	36.80	0.930	0.290	0.22	0.997
FAMW	1.5	0.48	0.940	0.026	0.970	4.28	0.970	0.160	0.76	0.995
	7.4	1.39	0.980	0.046	0.990	10.90	0.990	0.290	0.89	0.998
FAMD	1.5	0.96	0.950	0.034	0.980	9.30	0.980	0.190	0.82	0.996
	7.4	1.71	0.990	0.057	0.990	13.20	0.990	0.330	0.92	0.999

First-order model [ln(1 − Qt/Q∞) = − k1t]

Q∞ is the maximum amount of drug released, and k1 is the first-order release constant. This model assumes that the release rate depends on the amount of drug remaining in the carrier. The free folic acid formulation showed an excellent fit to the first-order model at both pH values (R^2^ = 0.999 and 0.997), confirming its concentration-dependent burst release. The FAMW and FAMD formulations also demonstrated good agreement with this model, with R^2^ values between 0.970 and 0.990. Their first-order release constants increased with pH, from 0.026 to 0.046 h⁻^1^ for FAMW and from 0.034 to 0.057 h⁻^1^ for FAMD, indicating enhanced release kinetics at pH 7.4 (Table 3). These findings suggest that concentration-dependent release contributes to the overall release behavior, particularly during the initial stages of drug release.

Higuchi model (Qt = kH√t)

kH is the Higuchi dissolution constant. The Higuchi model describes diffusion-controlled drug release from porous matrices. The FAMW and FAMD formulations exhibited excellent agreement with this model, particularly at pH 7.4 (R^2^ = 0.990 for both formulations), indicating that diffusion through the mesoporous MCM-41 channels was the dominant release mechanism. The Higuchi constants increased from 4.28 to 10.90 h^−1/2^ for FAMW and from 9.30 to 13.20 h^−1/2^ for FAMD with increasing pH, demonstrating enhanced diffusion under physiological conditions. The consistently higher Higuchi constants for FAMD compared with FAMW indicate faster diffusion and more efficient drug release from this formulation. In contrast, the free folic acid formulation exhibited lower correlation coefficients (R^2^ = 0.930–0.940), reflecting its immediate dissolution rather than diffusion-controlled release (Table 3).

Korsmeyer–Peppas model (Mt/M∞ = kKPtn)

Mt/M∞ represents the fraction of drug released at time *t*, kKP is the release rate constant, and *n* is the release exponent that characterizes the release mechanism. This model was applied to the initial stage of drug release (typically ≤ 60% cumulative release) to further elucidate the release mechanism. The FAMW and FAMD formulations showed excellent agreement with the Korsmeyer–Peppas model (R^2^ = 0.995–0.999). The release exponent (*n*) ranged from 0.76 to 0.89 for FAMW and from 0.82 to 0.92 for FAMD, indicating anomalous (non-Fickian) transport, in which drug release is governed by a combination of diffusion through the mesoporous network and drug–carrier interactions/desorption processes. The higher *n* values observed for FAMD suggest a greater contribution of matrix relaxation and interfacial interactions during release. Although the free folic acid formulation also produced high R^2^ values (0.997–0.998), its very low release exponent (*n* = 0.18–0.22) reflects the rapid burst release rather than a true diffusion-controlled mechanism, and therefore the Korsmeyer–Peppas model has limited mechanistic significance for this formulation (Table 3).

Overall, the kinetic analysis demonstrates that drug release from the MCM-41-based formulations is governed by multiple mechanisms. The poor fit of the free folic acid formulation to the zero-order model and its excellent fit to the first-order model confirm its rapid concentration-dependent dissolution. In contrast, the FAMW and FAMD formulations exhibited excellent agreement with both the Higuchi and Korsmeyer–Peppas models, demonstrating that folic acid release is predominantly diffusion-controlled with additional contributions from drug–carrier interactions. The consistently higher release rate constants obtained for FAMD in all kinetic models (k0, k1, kH, and kKP), together with its higher release exponent (*n*), indicate faster diffusion and more favorable release kinetics than FAMW, particularly at pH 7.4. These findings confirm that the drug loading medium and the resulting host–guest interactions play a critical role in modulating folic acid release from MCM-41 mesoporous silica.

### Cellular uptake profiles

**Fig. 6:**
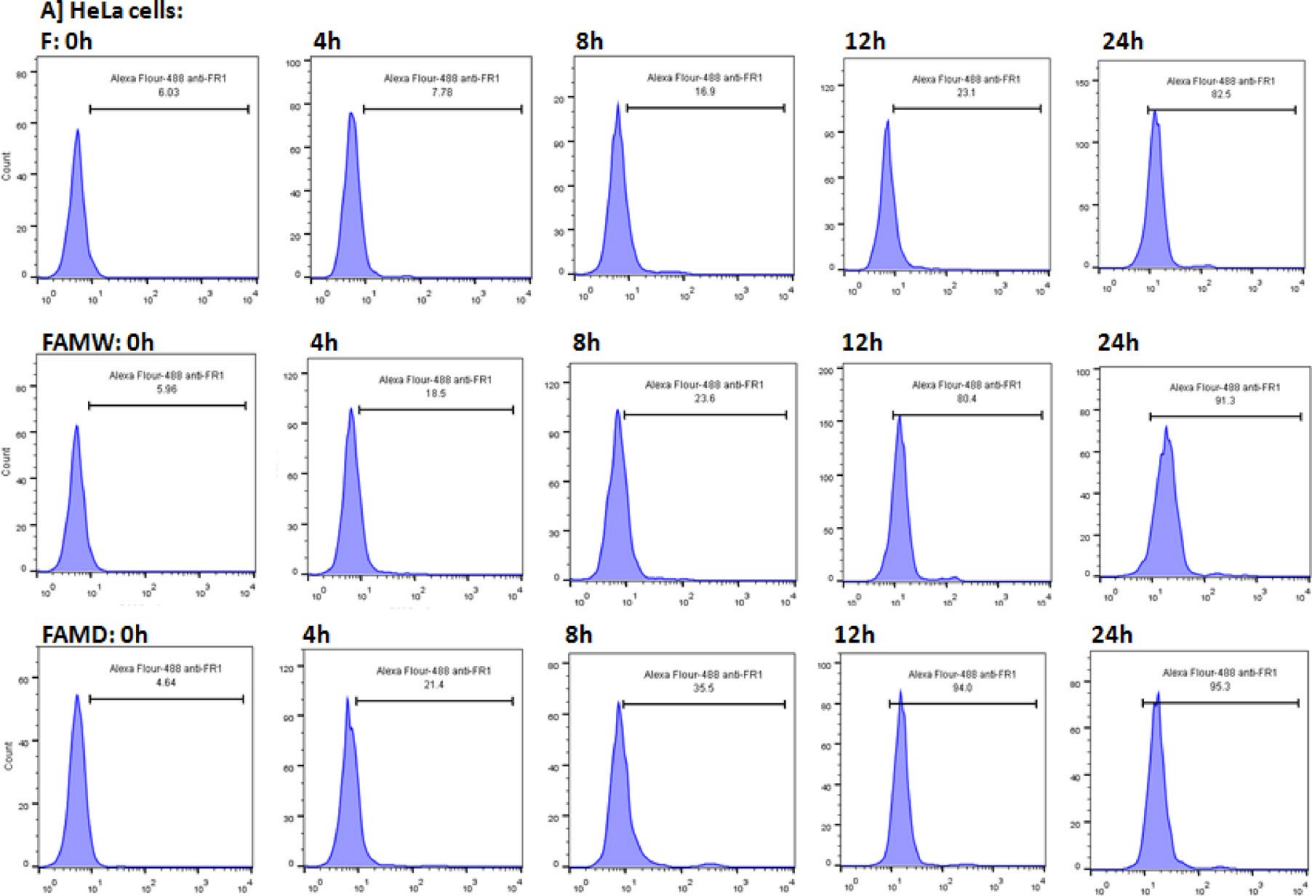

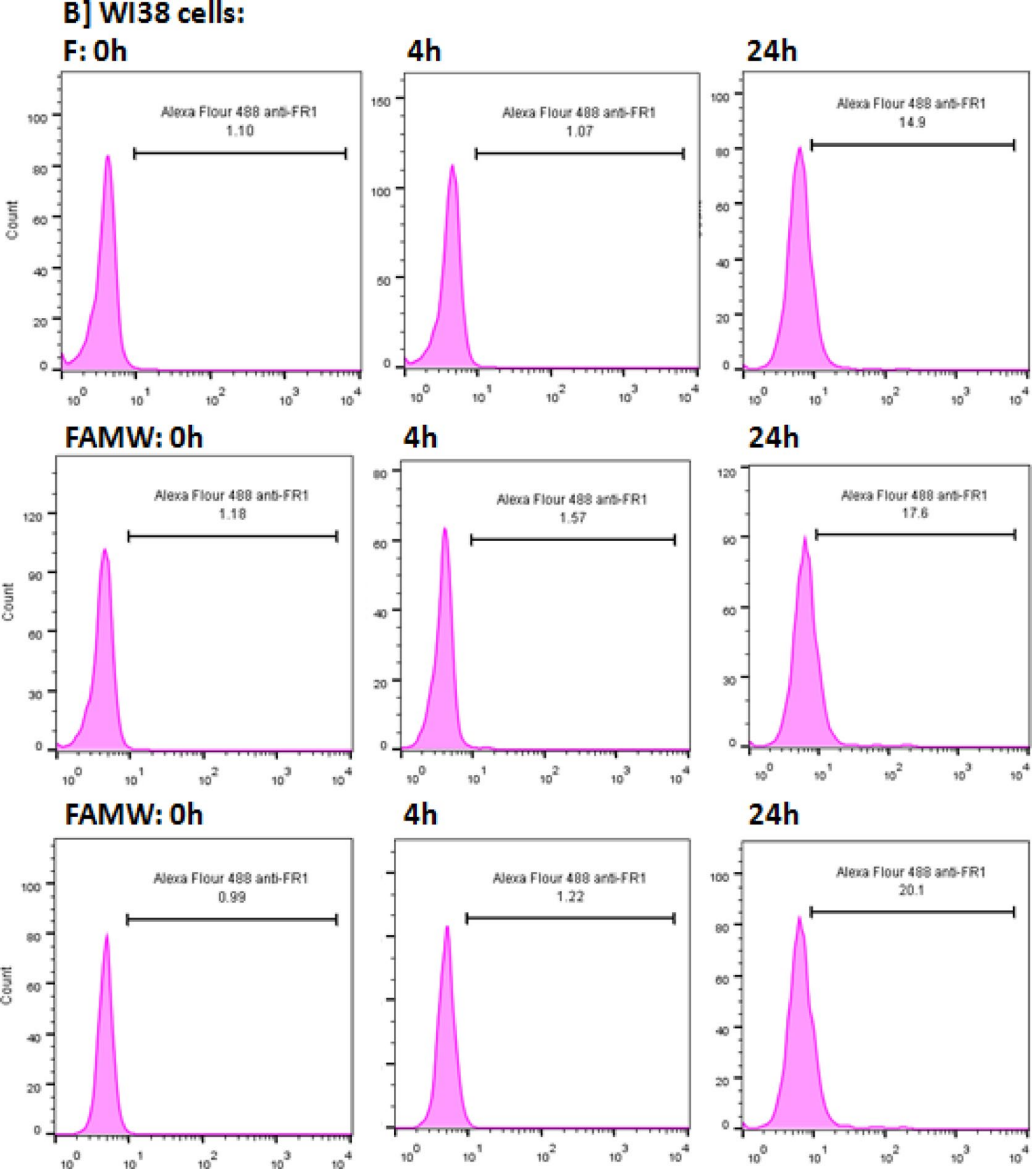
Cellular uptake of F, FAMW, and FAMD on HeLa cells (**A**) and WI38 cells (**B**).

The cellular uptake profiles of the formulations demonstrated a pronounced effect of FA-mediated targeting in HeLa cells (Fig. 6A). FA is consistently defined as a surface-conjugated targeting ligand responsible for folate receptor-mediated cancer cell internalization. At 0 h, all formulations exhibited comparable baseline fluorescence values ranging from 4.64% to 6.03%. The F showed a gradual increase in cellular uptake over time, reaching 7.78%, 16.9%, 23.1%, and 82.5% at 4, 8, 12, and 24 h, respectively.

In contrast, FA-modified formulations exhibited significantly enhanced uptake at earlier time points. FAMW showed uptake values of 18.5%, 23.6%, 80.8%, and 91.3% at 4, 8, 12, and 24 h, respectively. The highest uptake was observed for FAMD, which reached 21.4%, 35.5%, 94.0%, and 95.3% at the corresponding time points. These findings indicate that FA loaded MCM-14 substantially enhanced cellular internalization in FRα-overexpressing HeLa cells, with FAMD displaying the most efficient uptake.

In WI-38 cells (Fig. 6B), cellular uptake was markedly lower than that observed in HeLa cells at all-time points, although a moderate time-dependent increase was detected after 24 h. The F showed uptake values of 1.10%, 1.07%, and 14.9% at 0, 4, and 24 h, respectively. Similarly, FAMW exhibited uptake values of 1.18%, 1.57%, and 17.6%, while FAMD showed values of 0.99%, 1.22%, and 20.1% at the corresponding time points. Despite the increase at 24 h, uptake levels remained substantially lower than those observed in HeLa cells, particularly for the folate-targeted formulations.

### Cytotoxicity and safety profiles

**Fig. 7:**
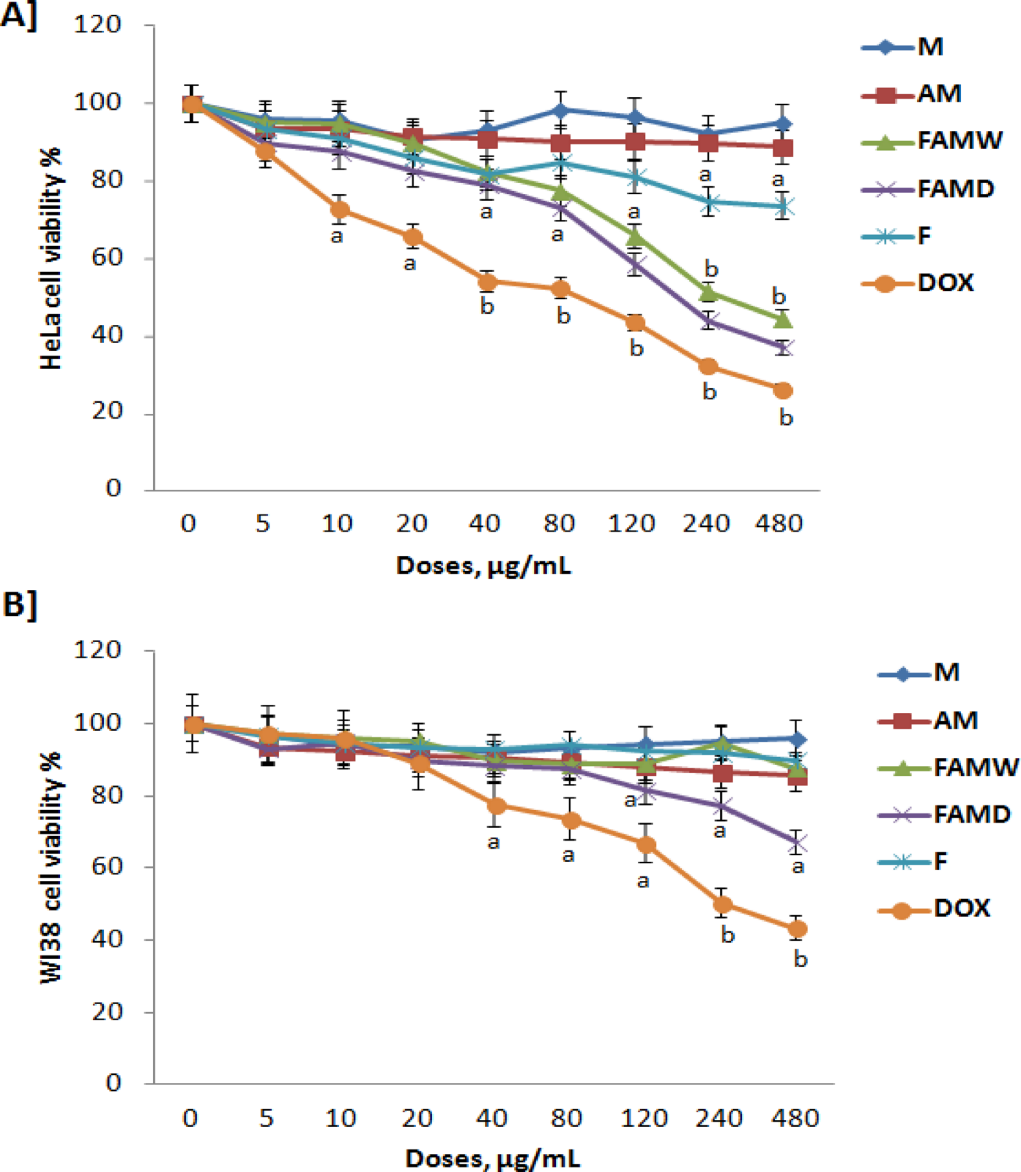

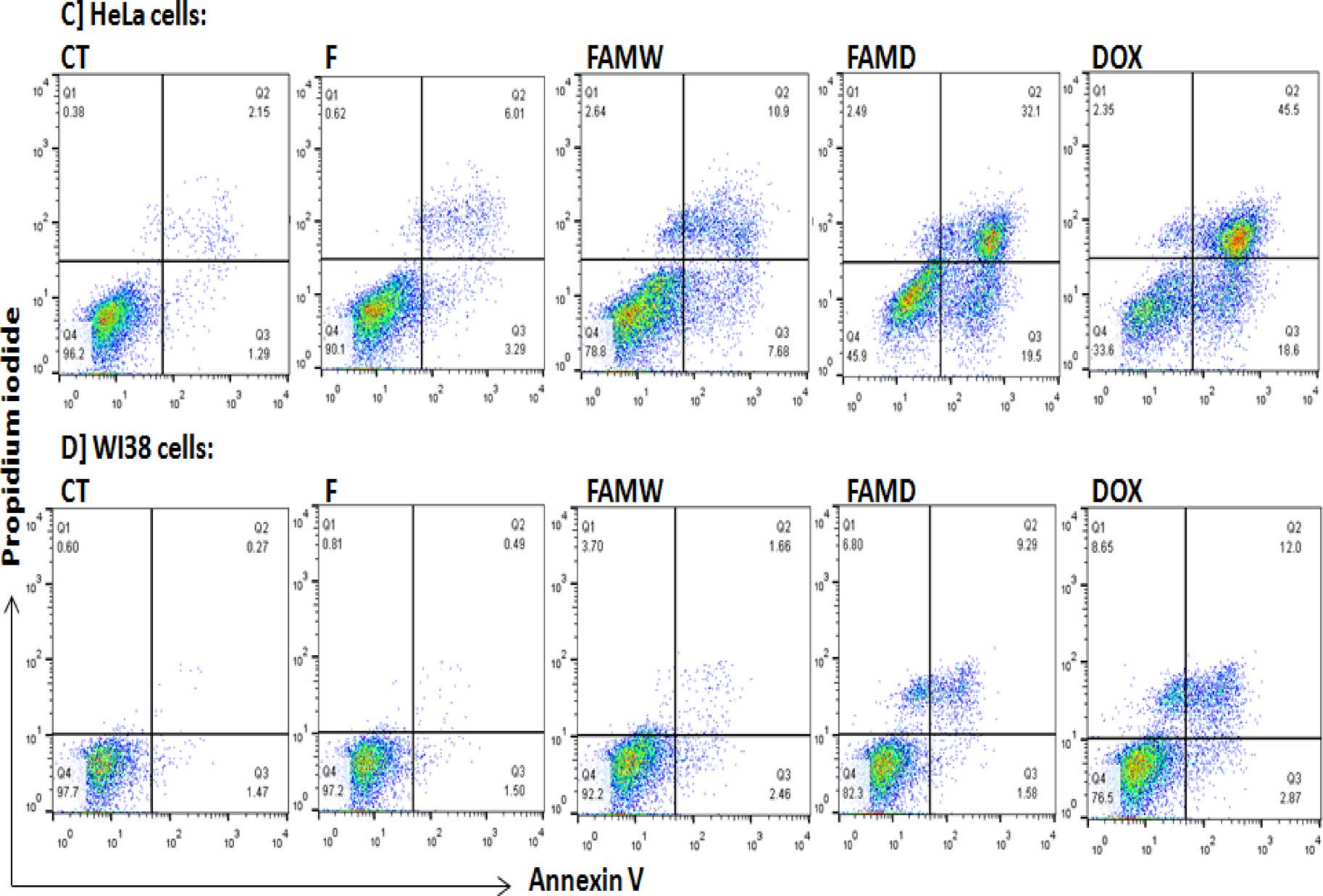
Cytotoxicity-based MTT assay (**A**, **B**) and apoptosis-based flow cytometry (**C**, **D**) of the different folic acid formulas against cervical HeLa cancerous cells and normal WI38 cells.

The pure MCM-41 (M) exhibited negligible cytotoxicity against cervical HeLa cancer cells, confirming its excellent biocompatibility. Functionalization with amino groups (AM) induced only minor, non-significant cytotoxic effects, resulting in a maximum cell death of approximately 11.2% at 480 µg/mL. Treatment with folic acid (F), which in this study is consistently considered as the loaded bioactive anticancer agent, showed a very low cytotoxic response, with a maximum cell death of approximately 26.5% at 480 µg/mL and an undetectable IC₅₀ value (Fig. 7).

In contrast, the drug-loaded nanocarrier systems FAMW and FAMD exhibited dose-dependent cytotoxicity against cancerous HeLa cells. At low concentrations (up to 40 µg/mL), both formulations showed minimal effects on cell viability; however, at higher concentrations (80–480 µg/mL), cytotoxicity increased significantly, reaching 55.7% and 63.1% cell death at 480 µg/mL for FAMW and FAMD, respectively. The IC₅₀ values were determined to be 82.99 µg/mL for FAMW and 77.73 µg/mL for FAMD, confirming the higher potency of FAMD. Notably, the IC₅₀ of FAMD was slightly higher than that of the standard chemotherapeutic drug doxorubicin (64.02 µg/mL), which induced the highest cytotoxicity with approximately 73.8% cell death at 480 µg/mL (Fig. 7A).

It should be noted that the concentration of doxorubicin (DOX) used as the positive control in the present study was intentionally selected to match the concentration range employed for the investigated nanocarrier formulations, thereby enabling a direct comparison of their cytotoxic responses under identical experimental conditions. This concentration is higher than those commonly used in standard in vitro cytotoxicity studies and was not intended to represent the typical therapeutic or physiological concentration of DOX. Therefore, the observed cytotoxicity of the positive control should be interpreted solely within the context of this comparative experimental design.

To test the safety of the proposed formulas FAMW and FAMD, normal WI38 cells were utilized (Fig. 7B). Firstly, all tested formulas (M, AM, F, FAMW, and FAMD) recorded untestable IC_50_ values against normal WI38 cells. Noting that, FAMD was non- significant cytotoxic against normal WI38 cells at low and moderate concentrations till 120 µg/mL, but the cytotoxicity of FAMD was slightly increased at high concentration (480 µg/mL), reached 32.6% cell death. Thus, we may use FAMD in low and moderate concentrations in the further studies to avoid tackling normal cells and to be specific against cancerous cells. Treatment of WI38 normal cells with DOX caused significant cytotoxicity, with approximate 56.60% cell death as maximum at 480 µg/mL concentration of DOX. The last observation demonstrated that DOX therapy was not selective.

Folic acid receptors are expressed in a variety of cancer cells^46^. Thus, folic acid-based systems performs better than folic acid-free systems on cancerous HeLa cells and not affecting normal WI38 cells^46^. These observations were in consistence with our data^51^.

Overall, folic acid alone is not sufficient to induce significant cytotoxic effects. In contrast, both FAMW and FAMD nanocarriers showed negligible cytotoxicity at low concentrations (≤ 40 µg/mL), while a marked increase in anticancer activity was observed at higher concentrations (≥ 80 µg/mL), with the most pronounced effects at 240–480 µg/mL. This confirms that the therapeutic effect is dose-dependent and primarily driven by nanocarrier-mediated delivery rather than the free drug.

Notably, the AM-functionalized nanocarrier showed slightly enhanced cytotoxicity compared to bare MCM-41. This improvement can be attributed to the introduction of surface amino groups, which increase positive surface charge, enhance electrostatic interaction with negatively charged cancer cell membranes, and improve cellular internalization. Additionally, amino functionalization may facilitate stronger hydrogen bonding and improved drug loading efficiency, leading to better intracellular drug retention and release behavior. These combined effects contribute to the modest increase in anticancer activity observed for AM compared to pristine MCM-41.

### Apoptosis-based flow cytometric results

Flow cytometric analysis using Annexin V–PI staining was performed to evaluate the apoptotic effects of free folic acid (F), FAMW, FAMD, and doxorubicin (DOX) on HeLa cancer cells and WI38 normal fibroblast cells. The results were expressed in terms of the viable (unapoptotic) population and the corresponding apoptotic fraction (Fig. 7C, D).

In HeLa cells, the untreated control group exhibited a high viable population of 96.2%, indicating minimal spontaneous apoptosis. Treatment with free folic acid resulted in a slight reduction in viability to 90.1%, corresponding to a modest increase in apoptotic cells. A more pronounced effect was observed with the nanocarrier systems, where FAMW reduced the viable cell population to 78.8%, indicating a substantial increase in apoptotic induction. Notably, FAMD showed a strong cytotoxic effect, decreasing the viable population further to 45.9%, which reflects a major shift toward apoptotic cell death. Doxorubicin, as a reference chemotherapeutic agent, produced the highest level of apoptosis, reducing viability to 33.6%. Overall, the apoptotic fraction followed the order DOX > FAMD > FAMW > F > control, indicating a clear enhancement in apoptosis induction with the formulated systems, particularly FAMD (Fig. 7C).

In contrast, WI38 normal fibroblast cells exhibited significantly higher resistance to all treatments. The untreated control showed 97.7% viable cells, confirming normal cell health. Free folic acid maintained a similarly high viability of 97.2%, while FAMW slightly reduced viability to 92.2%. FAMD showed a moderate decrease to 82.3%, indicating limited apoptotic induction in normal cells. Doxorubicin caused the strongest effect among the tested treatments, reducing viability to 76.5%, though still considerably higher than that observed in cancer cells. These results indicate a clear selectivity of the formulations toward cancer cells, particularly for FAMD, which induced strong apoptosis in HeLa cells while maintaining comparatively higher viability in WI38 cells (Fig. 7D).

To better interpret the apoptotic response, the apoptotic-to-viable cell ratio demonstrates the selectivity of cytotoxicity. In HeLa cells, this ratio increased markedly from minimal levels in the control group to progressively higher values in treated groups, reaching its maximum in DOX and FAMD, confirming strong apoptosis induction. In WI38 cells, the the apoptotic-to-viable cell ratios remained low across all treatments, particularly for F and FAMW, indicating low off-target toxicity. Even though FAMD induced higher apoptosis in WI38 compared to FAMW, the level remained significantly lower than that observed in HeLa cells, highlighting a favorable therapeutic window.

### Dose-dependent impact on stress mediators

**Fig. 8:**
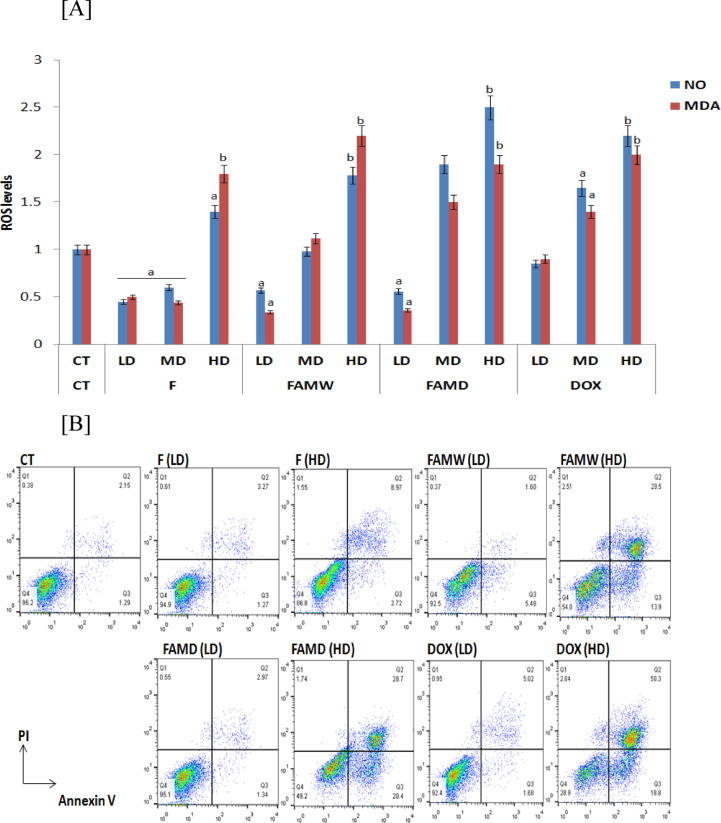
Dose-dependent impact on Stress mediators (**A**) and apoptosis (**B**) against cervical HeLa cancerous cells.

Reactive oxygen species (ROS) markers, including nitric oxide (NO) and malondialdehyde (MDA), were evaluated to assess oxidative stress-mediated cytotoxicity. In HeLa cells, a clear dose-dependent dual response was observed across folic acid (F), FAMW, FAMD, and doxorubicin (DOX). At low doses (LD), all formulations exhibited antioxidant behavior, reducing oxidative stress markers below control levels. The strongest reduction was observed with folic acid for NO (0.45-fold) and MDA (0.50-fold), while FAMD also significantly decreased NO (0.56-fold) and MDA (0.36-fold), indicating strong antioxidant potential at low concentration. At medium doses (MD), a formulation-dependent response was observed, where F showed moderate NO reduction (0.60) and slight MDA decrease (0.44), whereas FAMW showed a shift toward increased oxidative stress for NO (0.98) with elevated MDA (1.12). In contrast, FAMD clearly promoted oxidative stress at MD, increasing NO (1.9-fold) and MDA (1.5-fold), while DOX showed moderate increases for both markers (Fig. 8A).

At high doses (HD), all treatments induced strong oxidative stress. F increased NO and MDA levels to 1.4 and 1.8-fold, respectively, while FAMW further elevated them to 1.78 (NO) and 2.2 (MDA). FAMD showed the most pronounced effect, significantly increasing NO (2.5-fold) and MDA (1.9-fold), confirming its potent pro-oxidant activity at higher concentration. DOX also elevated oxidative stress markers (2.2-fold NO and 2.0-fold MDA), consistent with its known cytotoxic mechanism (Fig. 8A).

This dose-dependent dual effect suggests redox modulation, where the formulations act as antioxidants at low doses and pro-oxidants at higher doses. The increased oxidative stress at high concentrations likely exceeds cellular antioxidant defenses, leading to mitochondrial damage and apoptosis activation. The stronger effect of FAMD may be due to improved cellular uptake via folate targeting and enhanced intracellular interaction. Such ROS-mediated cytotoxicity is a well-established mechanism in nanomaterial-based cancer therapy^52,53^.

#### Dose-dependent impact on apoptosis

The untreated control group showed a high viable (unapoptotic) population of 96.2%, indicating normal cell survival with minimal spontaneous apoptosis (Fig. 8B).

At low dose (LD), all treatments showed only mild reductions in cell viability. Free folic acid (F-LD) maintained a high viable population of 94.9%, while FAMW-LD and DOX-LD showed similar values of 92.5% and 92.4%, respectively, indicating limited apoptosis induction at lower concentrations. These results suggest that low-dose exposure primarily preserves cell viability with minimal activation of apoptotic pathways (Fig. 8B).

In contrast, at high dose (HD), a marked decrease in viable cells was observed, indicating strong apoptosis induction. Folic acid (F-HD) reduced viability to 86.8%, while FAMW-HD showed a more pronounced effect with 54% viable cells. The most significant reduction was observed with FAMD-HD, where viability decreased to 49.2%, indicating substantial apoptosis activation. DOX-HD produced the strongest cytotoxic effect among all treatments, reducing viable cells to 28.9%, reflecting extensive apoptosis (Fig. 8B).

Overall, the flow cytometry results demonstrate a clear dose-dependent apoptotic response in HeLa cells, where high doses significantly enhance programmed cell death. Among the tested formulations, FAMD exhibited strong pro-apoptotic activity, while doxorubicin remained the most potent inducer of apoptosis, confirming the effectiveness of the formulations in triggering apoptosis in a concentration-dependent manner. Importantly, the ROS (NO and MDA) and flow cytometry results together confirm a clear dose-dependent oxidative stress–apoptosis relationship in HeLa cells.

#### Antioxidant enzymes

**Fig. 9:**
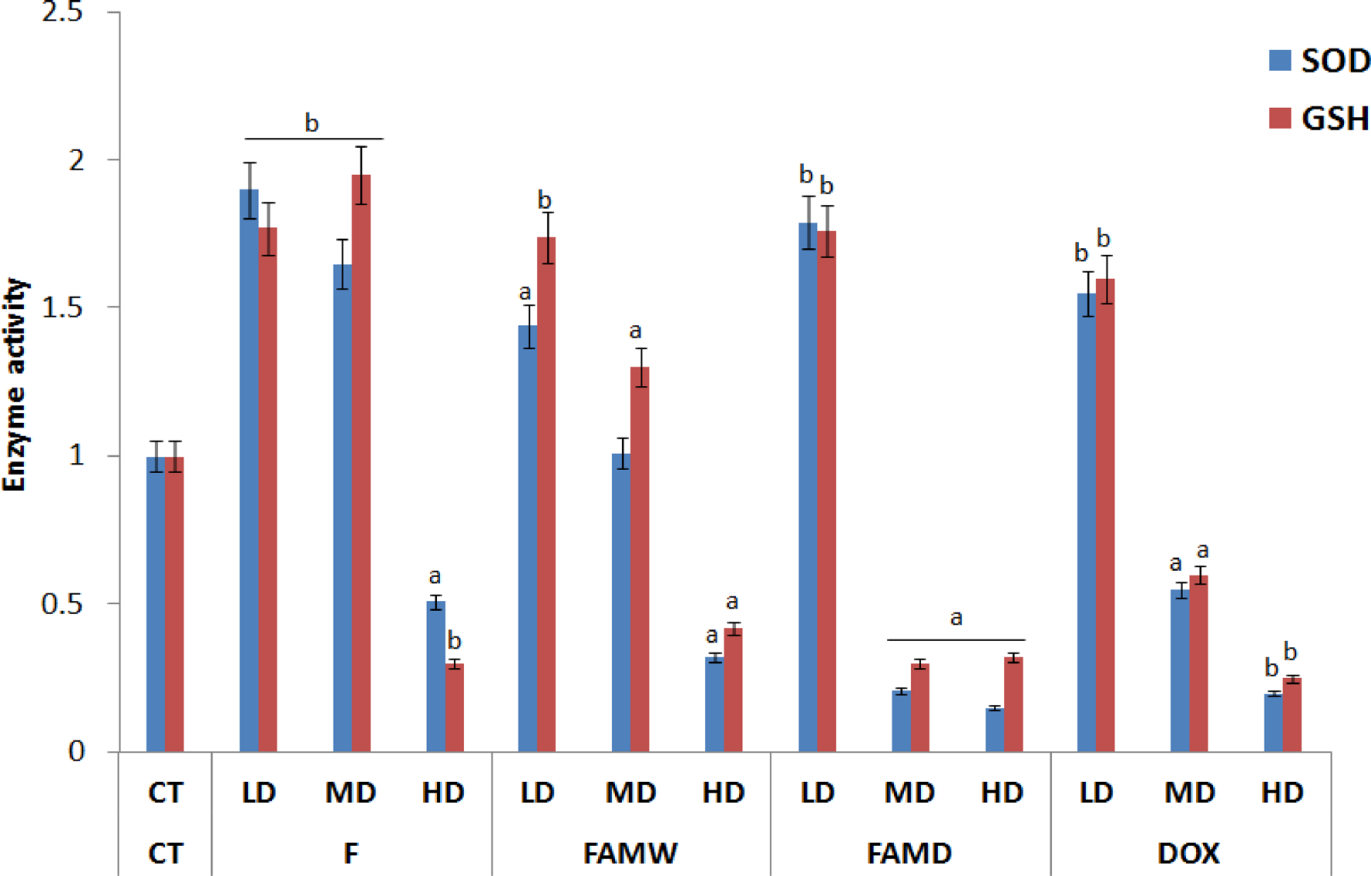
Enzyme activity. Superoxide dismutase (SOD) and glutathione (GSH) in HeLa cells (*n* = 3).

Antioxidant enzymes **(**Fig. 9), including superoxide dismutase (SOD) and glutathione (GSH), were evaluated in HeLa cells to assess redox regulation under different treatment doses. At low doses (LD), all formulations enhanced antioxidant defense systems, with folic acid (F) showing the strongest increase in SOD (1.9-fold) and GSH (1.77-fold), followed by FAMW (SOD 1.44-fold, GSH 1.74-fold) and FAMD (SOD 1.79-fold, GSH 1.76-fold), indicating an overall antioxidant response. At moderate doses (MD), a formulation-dependent shift was observed, where F maintained elevated antioxidant activity (SOD 1.65, GSH 1.95), while FAMW showed partial enhancement (SOD 1.01, GSH 1.3), and FAMD already began to suppress antioxidant defenses (SOD 0.21, GSH 0.30). At high doses (HD), all treatments significantly reduced antioxidant enzyme levels, confirming strong oxidative stress induction. The most pronounced decrease was observed with FAMD, where SOD and GSH dropped to 0.15 and 0.32, respectively, followed by DOX (SOD 0.20, GSH 0.25) and FAMW (SOD 0.32, GSH 0.42), while free folic acid showed a moderate reduction (SOD 0.51, GSH 0.30).

Overall, these results demonstrate a clear dose-dependent dual effect, where low doses enhance antioxidant defenses, whereas high doses suppress SOD and GSH activities due to excessive ROS accumulation and oxidative stress–mediated apoptosis, with FAMD exhibiting the strongest pro-oxidant effect. These findings support the role of oxidative stress imbalance in triggering apoptosis in cervical cancer cells^52,54^.

At low doses, the FAMW and FAMD systems appear to induce a mild redox buffering effect, as evidenced by decreased NO and MDA levels alongside increased SOD and GSH activity. This response can be interpreted as an initial cellular adaptive antioxidant mechanism, where the nanocarrier-mediated delivery of folic acid induces sub-lethal stress that activates endogenous antioxidant defenses rather than overwhelming them. In this regime, ROS production remains within the physiological tolerance range, allowing cancer cells to partially counterbalance oxidative stress through upregulation of antioxidant enzymes and maintenance of redox homeostasis.

Mechanistically, this low-dose behavior may be attributed to limited intracellular accumulation of the nanocarriers, resulting in insufficient ROS amplification to surpass the apoptotic threshold. As a consequence, cells engage survival pathways and antioxidant upregulation to restore redox equilibrium. However, as the dose increases, intracellular accumulation of the nanocarriers is enhanced, leading to excessive ROS generation that overwhelms the cellular antioxidant system. This shift disrupts redox homeostasis, suppresses SOD and GSH, and drives oxidative damage, ultimately triggering mitochondrial dysfunction and apoptosis.

Thus, the system exhibits a dose-dependent redox switch, where low concentrations promote an adaptive antioxidant response, whereas higher concentrations induce oxidative stress–mediated cytotoxicity. This biphasic behavior highlights the critical role of dose control in determining whether the system elicits cellular survival or apoptotic signaling in cancer cells.

#### Bax and Bcl-2 expression analysis

**Fig. 10:**
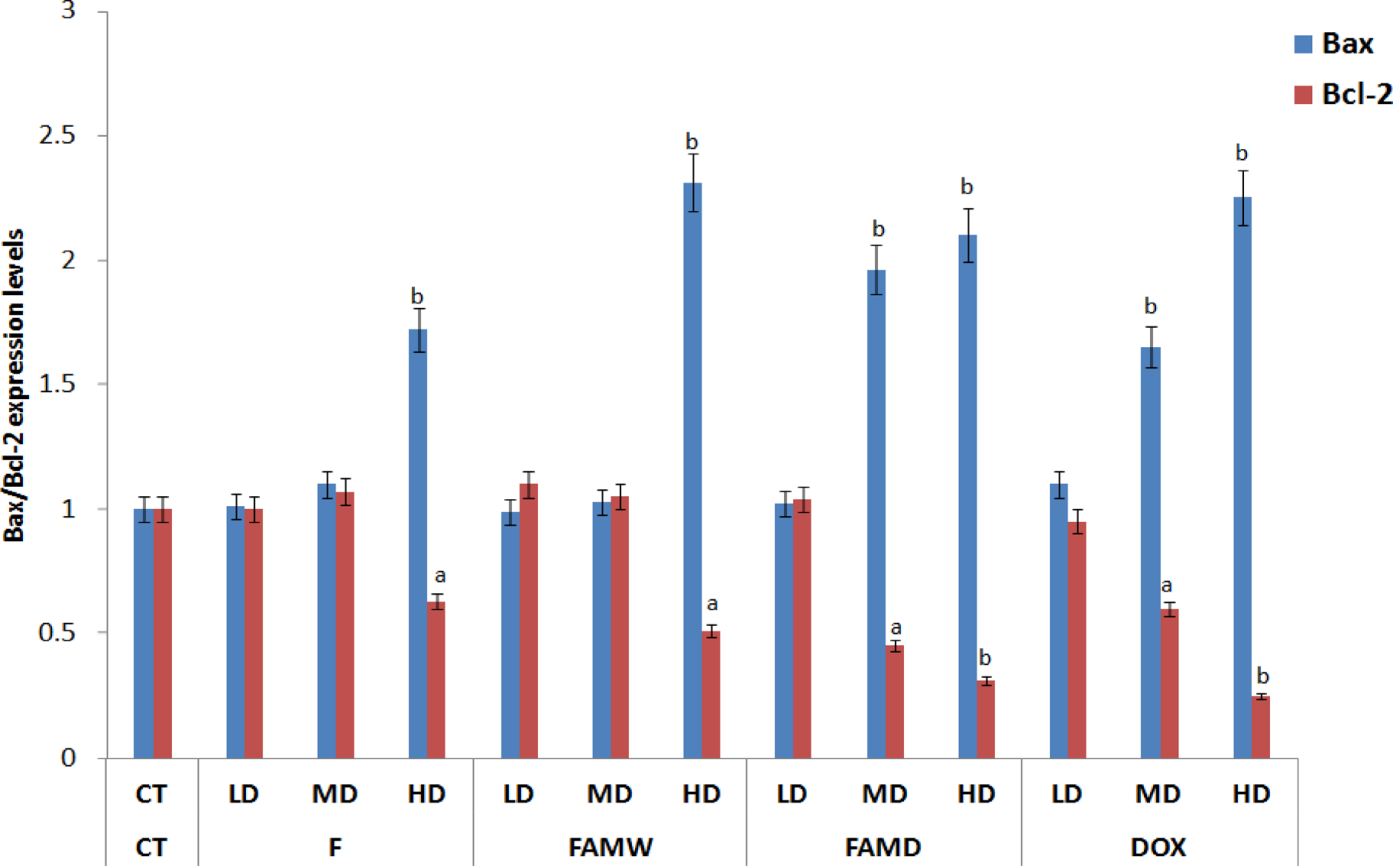
Apoptotic protein expression levels. Bax and Bcl-2 expression levels (*n* = 3).

The expression levels of the apoptotic regulatory proteins Bax (pro-apoptotic) and Bcl-2 (anti-apoptotic) were evaluated to understand the mitochondrial pathway activation in HeLa cells under different treatments. Bax showed a dose-dependent upregulation, while Bcl-2 exhibited a corresponding downregulation, indicating activation of intrinsic apoptosis (Fig. 10).

At low dose (LD), Bax expression showed only slight changes compared to control (1.01 for F, 0.99 for FAMW, and 1.02 for FAMD), while Bcl-2 remained nearly unchanged or slightly elevated (1.00 for F, 1.10 for FAMW, 1.04 for FAMD), suggesting minimal apoptotic activation at low concentrations.

At moderate dose (MD), a clearer shift toward apoptosis was observed. Bax increased to 1.10 (F), 1.03 (FAMW), and 1.96 (FAMD), while Bcl-2 decreased to 1.07 (F), 1.05 (FAMW), and 0.45 (FAMD), indicating strong mitochondrial pathway activation particularly in FAMD-treated cells.

At high dose (HD), Bax expression reached its maximum levels in all formulations, especially in FAMD (2.10) and DOX (2.25), followed by F (1.72) and FAMW (2.31). In contrast, Bcl-2 was markedly suppressed, reaching 0.63 (F), 0.51 (FAMW), 0.31 (FAMD), and 0.25 (DOX). These results confirm strong induction of apoptosis at higher doses, with FAMD showing effects comparable to doxorubicin.

The Bax/Bcl-2 ratio is a key indicator of mitochondrial-mediated apoptosis, where higher values reflect stronger apoptotic signaling. At low dose, all treatments showed ratios close to 1, indicating balanced survival and apoptotic signaling. At moderate dose, a pronounced increase was observed, especially for FAMD (4.36), suggesting strong activation of apoptotic pathways, while F and FAMW showed only slight increases. At high dose, all formulations induced significant apoptosis, with a marked rise in Bax/Bcl-2 ratios. DOX exhibited the highest ratio (9.00), confirming its strong cytotoxic potency. Notably, FAMD also showed a very high ratio (6.77), demonstrating potent pro-apoptotic activity, significantly higher than F (2.73) and FAMW (4.53) (Table 4). This confirms that FAMD effectively shifts the balance toward apoptosis by upregulating Bax and suppressing Bcl-2, thereby strongly activating the intrinsic mitochondrial apoptotic pathway.

**Table 4:** Bax/Bcl-2 ratio.

Treatment	LD	MD	HD
F	1.01	1.03	2.73
FAMW	0.90	0.98	4.53
FAMD	0.98	4.36	6.77
DOX	1.16	2.75	9.00

An increased Bax/Bcl-2 ratio is a key indicator of activation of the intrinsic (mitochondrial) apoptotic pathway. The observed upregulation of Bax together with downregulation of Bcl-2 suggests mitochondrial membrane destabilization, leading to cytochrome c release and subsequent caspase activation. The strongest effect observed in FAMD indicates enhanced pro-apoptotic potential, likely due to improved folate receptor-mediated uptake and higher intracellular drug accumulation. These findings confirm that the synthesized folic acid-based nanocarriers effectively trigger mitochondria-dependent apoptosis in cervical cancer cells^55,56^.

#### PI3K/AKT signalling pathway

**Fig. 11:**
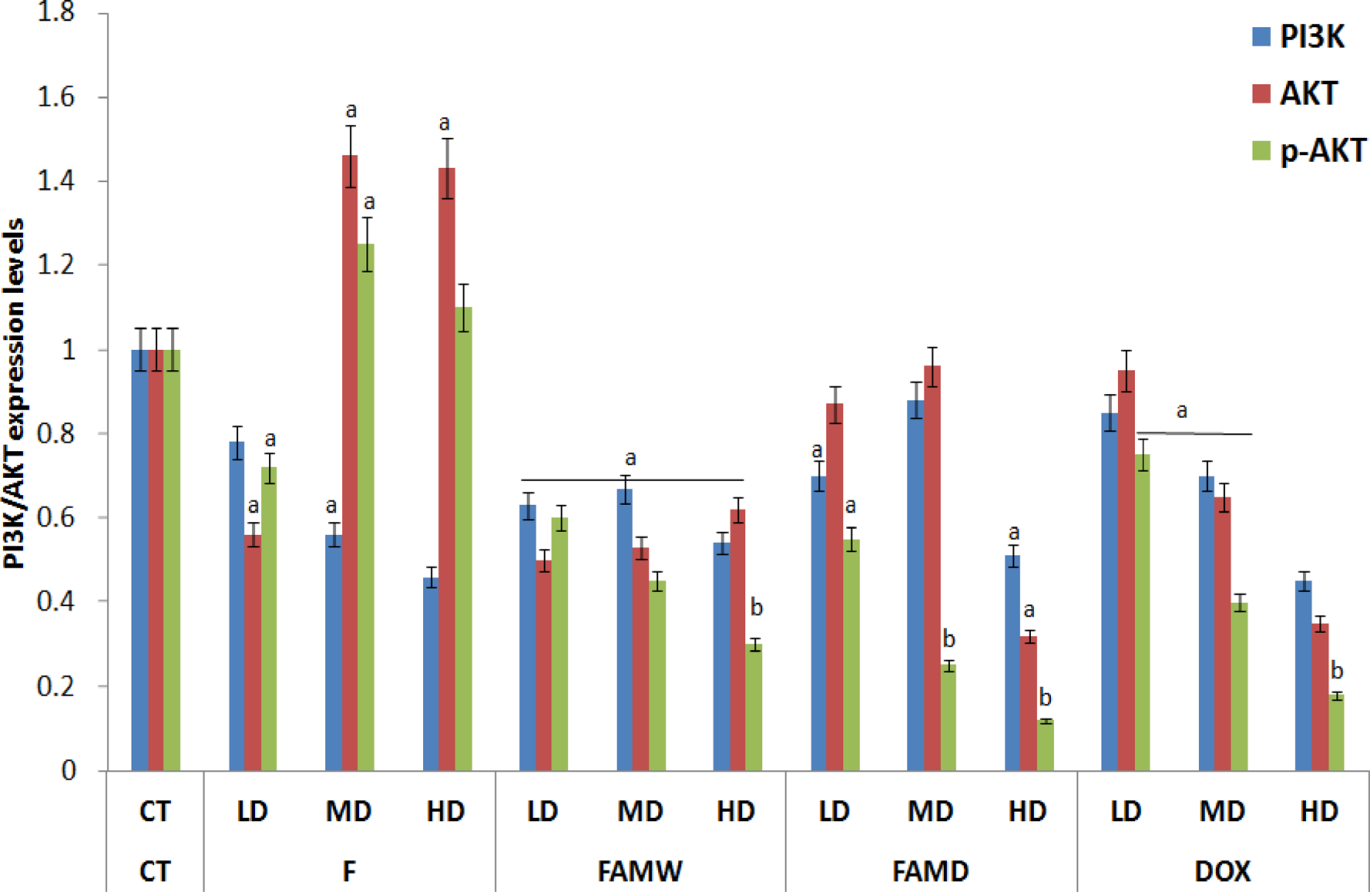
Survival protein expression levels. PI3K and AKT expression levels (*n* = 3).

The PI3K/AKT signaling pathway, a central regulator of cell survival and anti-apoptotic responses, was markedly modulated in HeLa cells following treatment with folic acid (F), FAMW, and FAMD in a dose-dependent manner. PI3K expression showed a consistent overall downregulation across treatments compared to control (1.0), with values decreasing particularly at high dose for all formulations, reaching 0.46 (F), 0.54 (FAMW), and 0.51 (FAMD), indicating effective inhibition of upstream survival signaling. Even at low and moderate doses, PI3K levels were generally suppressed or maintained below control, confirming early pathway interference (Fig. 11).

AKT expression showed a more complex and dose-dependent response. Free folic acid (F) significantly reduced AKT at low dose (0.56), but moderate and high doses led to increased expression (1.46 and 1.43), suggesting activation of compensatory survival signaling at higher concentrations. In contrast, FAMW demonstrated a more stable inhibitory effect on AKT, with sustained downregulation across all doses (0.50, 0.53, and 0.62), indicating consistent suppression of downstream survival signaling. FAMD showed moderate AKT suppression at low dose (0.87), slight elevation at moderate dose (0.96), and strong inhibition at high dose (0.32), reflecting a dose-dependent switch toward pathway shutdown.

Importantly, p-AKT (the active phosphorylated form of AKT) exhibited the most pronounced and biologically relevant changes. In all formulations, p-AKT was strongly reduced in a dose-dependent manner, highlighting effective blockade of pathway activation. F showed moderate inhibition at low dose (0.72) and near-maintained activation at moderate and high doses (1.25 and 1.10), indicating limited control over pathway phosphorylation at higher concentrations. FAMW induced stronger suppression of p-AKT, decreasing it progressively from 0.60 (LD) to 0.45 (MD) and 0.30 (HD), confirming effective inhibition of AKT activation. The most significant effect was observed in FAMD, where p-AKT levels were sharply reduced to 0.55 (LD), 0.25 (MD), and 0.12 (HD), demonstrating potent inhibition of AKT phosphorylation and thus complete suppression of pathway activation at high dose.

Overall, the results clearly demonstrate that while PI3K is moderately suppressed across all systems, the decisive regulatory effect occurs at the level of AKT phosphorylation. The strong reduction of p-AKT, particularly in FAMW and most prominently in FAMD, confirms effective blockade of the PI3K/AKT survival pathway. This inhibition prevents downstream anti-apoptotic signaling and supports activation of mitochondrial apoptosis, consistent with Bax/Bcl-2 modulation and ROS-mediated cytotoxicity, confirming that FAMD exhibits the strongest pathway-targeted anticancer activity. These findings confirm that the synthesized nanocarriers induce apoptosis not only through oxidative stress but also via suppression of PI3K/AKT survival signaling^30,57^.

#### Correlation of FAMD-induced anticancer mechanism in HeLa Cells

FAMD treatment showed the most pronounced correlated biological response across all assays. At the level of apoptosis (Annexin V–PI flow cytometry), FAMD produced a strong increase in apoptotic cell population with a marked reduction in viable HeLa cells. This effect was accompanied by a significant downregulation of PI3K expression together with a marked decrease in p-AKT levels, indicating suppression of the PI3K/AKT survival signaling axis and reduced AKT activation. In parallel, FAMD induced elevated oxidative stress markers (NO and MDA) and depletion of antioxidant defenses (SOD and GSH), reflecting intracellular redox imbalance. This was further associated with an increased Bax/Bcl-2 ratio, indicating mitochondrial involvement in apoptosis. Collectively, these correlated changes—PI3K downregulation, p-AKT suppression, oxidative stress elevation, mitochondrial apoptotic shift, and increased apoptosis—represent an integrated response pattern associated with the strong cytotoxic effect of FAMD in HeLa cells.

## Conclusion

This study successfully developed a folic acid-loaded amino-functionalized MCM-41-derived silica nanocarrier (FAMD) as a potential targeted delivery system for cervical cancer therapy. Pure MCM-41 was synthesized, surface-functionalized with aminopropyl groups, and subsequently loaded with folic acid. Structural characterization confirmed that the ordered silica framework of MCM-41 was retained after functionalization and drug loading, while nitrogen adsorption–desorption analysis revealed a substantial reduction in the accessible surface area and pore volume (from 1100 m^2^/g for pure MCM-41 to 70.67, 53.10, and 35.40 m^2^/g for the AM, FAMW, and FAMD formulations, respectively), confirming successful incorporation of organic functionalities and folic acid within the silica framework. TEM observations further supported the preservation of the particle morphology and ordered structure following modification.

The FAMD formulation exhibited sustained and pH-responsive folic acid release, with higher release rates at pH 7.4 than at pH 1.5, demonstrating its potential for controlled drug delivery under physiological conditions. Kinetic analysis indicated that drug release from the functionalized formulations was predominantly diffusion-controlled, with additional contributions from drug–carrier interactions, as evidenced by the excellent agreement with the Higuchi and Korsmeyer–Peppas models. Compared with FAMW, FAMD exhibited faster release kinetics, reflecting differences in drug–carrier interactions and diffusion behavior.

Biological evaluation demonstrated that the developed silica-based nanocarrier exhibited selective cytotoxicity toward HeLa cervical cancer cells while maintaining comparatively low toxicity toward normal WI-38 cells. The formulation induced apoptosis through multiple mechanisms, including increased oxidative stress (elevated NO and MDA levels), depletion of antioxidant defenses (reduced SOD and GSH activities), an increased Bax/Bcl-2 ratio, and suppression of the PI3K/AKT signaling pathway. Collectively, these findings suggest that the developed functionalized MCM-41-derived silica nanocarrier is a promising platform for folic acid delivery and targeted anticancer applications.

### Limitations

Nevertheless, this study has some limitations. The biological evaluation was limited to in vitro experiments using a single cervical cancer cell line and one normal cell line, and therefore the therapeutic efficacy, biodistribution, pharmacokinetics, long-term biocompatibility, and systemic safety of the developed nanocarrier remain to be established in appropriate in vivo models. Furthermore, although the final FAMD formulation retained the ordered silica framework of the parent MCM-41 material, the substantial reduction in accessible surface area following functionalization and drug loading indicates that the accessible mesoporosity was markedly reduced. Future studies should therefore focus on optimizing the balance between surface functionalization and pore accessibility while evaluating the in vivo targeting efficiency and therapeutic performance of the developed nanocarrier.

## Data Availability

The datasets used and/or analyzed during the current study are available from the corresponding author on reasonable request.
